# Investigating the Impact of Low-Rate Error-Correction Codes on Device-Cloning and Reverse-Engineering Attacks on Physical Unclonable Functions

**DOI:** 10.3390/s26196247

**Published:** 2026-10-01

**Authors:** Sinan Yavuz, Edwin Naroska, Kai Daniel, Roman Obermaisser

**Affiliations:** 1 Department of Electrical Engineering and Computer Science, University of Siegen, 57076 Siegen, Germany; roman.obermaisser@uni-siegen.de; 2 Department of Electrical Engineering and Computer Science, Hochschule Niederrhein University of Applied Sciences, 47805 Krefeld, Germany; edwin.naroska@hs-niederrhein.de; 3 Institute of Measurement and Sensor Technology, University of Applied Sciences Ruhr West, 45479 Mülheim, Germany; kai.daniel@hs-ruhrwest.de

**Keywords:** physical unclonable functions (PUFs), security analysis, PUF reliability, cloning attack, reverse-engineering, lightweight security, PUF-based key generation, IoT

## Abstract

Securing Internet of Things (IoT) devices is important due to the rapid growth of smart devices and increasing demand for artificial intelligence (AI)-enabled functionality. However, IoT devices are often resource-constrained, making robust security measures challenging to implement. To address this issue, physical unclonable functions (PUFs) can be implemented on chips to generate device-specific signatures. Due to their lightweight structure, PUFs are suitable for resource-constrained IoT devices. However, PUFs are sensitive to environmental and operational variations, which can cause bit errors in the generated signatures and affect security applications. These errors can be corrected using error-correction codes (ECCs), enabling reliable signatures under varying conditions. This paper quantitatively investigates potential security risks associated with low-rate low-density parity-check (LDPC) codes in PUF-based applications. Two attack scenarios are analyzed using a modeled PUF. Our model-based results show that low-rate LDPC codes can significantly improve PUF reliability but may also introduce security risks. For the evaluated device-cloning attack scenario, the WiMAX LDPC code with a code rate of 1/2 and a soft-information length of K=288 resulted in a maximum success probability of 62.98% for reconstructing the secret response at an ambient temperature of 10 °C, demonstrating a potential security vulnerability for this configuration.

## 1. Introduction

Lightweight security measures to protect resource-limited Internet of Things (IoT) devices are an essential aspect. In addition, due to the rapid global growth and rising demand for artificial intelligence (AI)-enabled devices, this becomes more important [1,2]. However, this poses a significant challenge for the supply chain, the network (due to increased data transmissions), and the security mechanisms required to protect sensitive data against third parties [3]. The hardware structure of such IoT devices is typically based on conventional and lightweight chips that are developed for specific tasks and therefore do not provide a wide variety of functionality or high computational capabilities [1,3]. Due to these factors, protecting lightweight IoT devices poses a crucial challenge, especially when processing, receiving, and transmitting sensitive data in crucial applications [1] such as industry [2], healthcare [4,5], military [6,7], smart cities [8,9], or smart grids [10,11,12].

To address this issue, physical unclonable functions (PUFs) can be integrated and used in security applications to improve the security of lightweight IoT devices [13,14]. Silicon-based PUFs are hardware structures that can be implemented on FPGAs and ASICs and allow the generation of unique output signals, referred to as responses, to specific input signals, which are referred to as challenges. The combination of a challenge and the corresponding response is called a challenge-response pair (CRP) [15,16,17]. Depending on the structure and size of the PUF, hundreds or even millions of different CRPs can be generated [18]. The response generation is based on microscopic and uncontrollable variations in the fabrication process of the chip [19], such as variations in wire geometry or electrical characteristics in transistors [16]. These variations, together with environmental and operational conditions, lead to slight differences in the delay characteristics of individual transistors and wires. Therefore, in delay-based PUFs, the response is obtained based on signal propagation delays.

However, PUFs are sensitive to various environmental and operational conditions, such as ambient temperature variations, supply voltage fluctuations, electromagnetic interference, and aging effects [20]. These factors can impact the behavior of the PUF design. Especially during the regeneration of a response by using the corresponding challenge, bit errors can occur in the responses, affecting the reliability of the PUF design. To avoid this, error-correction codes (ECCs), which are typically used for correcting bit errors during data transmission over a noisy communication channel, can be combined with PUFs to correct bit errors and increase reliability [21,22,23,24,25,26]. However, a decrease in reliability can also be expected during operation due to hardware aging effects [27,28]. To correct bit errors of unreliable PUFs and increase the operational lifecycle of the PUF-based device, low-rate ECCs can be used, enabling high error-correcting performance, as presented in [20]. On the other hand, low-rate ECCs may pose additional vulnerabilities when used in PUF-based security applications. These ECCs contain a large amount of parity information, which may reveal crucial information about the response.

In this work, the security issues associated with low-rate ECCs in PUF-based applications are investigated. To this end, we improve and employ the PUF response model presented in [20] by incorporating the evaluated statistical parameters regarding bit stability and bit error characteristics of our proposed 2-4 DAPUF design from [18,29], which was tested on 110 FPGAs and evaluated using a large number of CRPs. The resulting simulated PUF design therefore generates soft responses with approximately the same statistical characteristics and is used as the PUF primitive in our system model for on-chip key generation. To investigate the impact of ECCs on security, the WiMAX (Worldwide Interoperability for Microwave Access) low-density parity-check (LDPC) codes with code rates of 1/2, 2/3, and 3/4 are utilized. For the security analysis, two threat models—a cloning attack and a reverse-engineering attack—are considered as representative attack scenarios for PUF-based systems. Therefore, for each threat model and each code rate, the probability of a successful attack is evaluated. In contrast to our previous work [20], which focused on the reliability analysis of PUF responses and the corresponding LDPC error-correction performance, this work focuses on the security implications of using low-rate LDPC codes in PUF-based applications. The previous PUF response model and the corresponding statistical PUF characteristics therefore provide the methodological basis for this work, while the security analysis based on the system model, the two considered threat models, and the quantitative evaluation of the corresponding attack success probabilities represent the main focus of this manuscript.

The contributions of this paper are to provide an overview of a PUF-based security application for memory encryption using soft responses, to introduce two threat models for the reconstruction of the secret PUF response, and to quantitatively investigate potential security risks associated with low-rate LDPC codes using a modeled PUF that approximates the statistical characteristics of an implemented PUF design. This highlights a potential trade-off between reliability and security when low-rate ECCs are used in PUF-based applications.

This paper is structured as follows: Section 2 introduces PUFs and presents related work on PUF reliability. In Section 3, preliminary considerations, the proposed system, additional model assumptions, and both threat models are discussed. Section 4 evaluates and discusses the model-based results of the security analysis. Finally, Section 5 concludes the paper and highlights our future research directions.

## 2. Background and Related Work

In this section, an introduction to delay-based PUFs and related work on PUF reliability regarding influencing factors for various conditions are briefly presented, highlighting the importance of error-correction mechanisms to improve PUF reliability.

### 2.1. Delay-Based Physical Unclonable Functions

**Figure 1:**
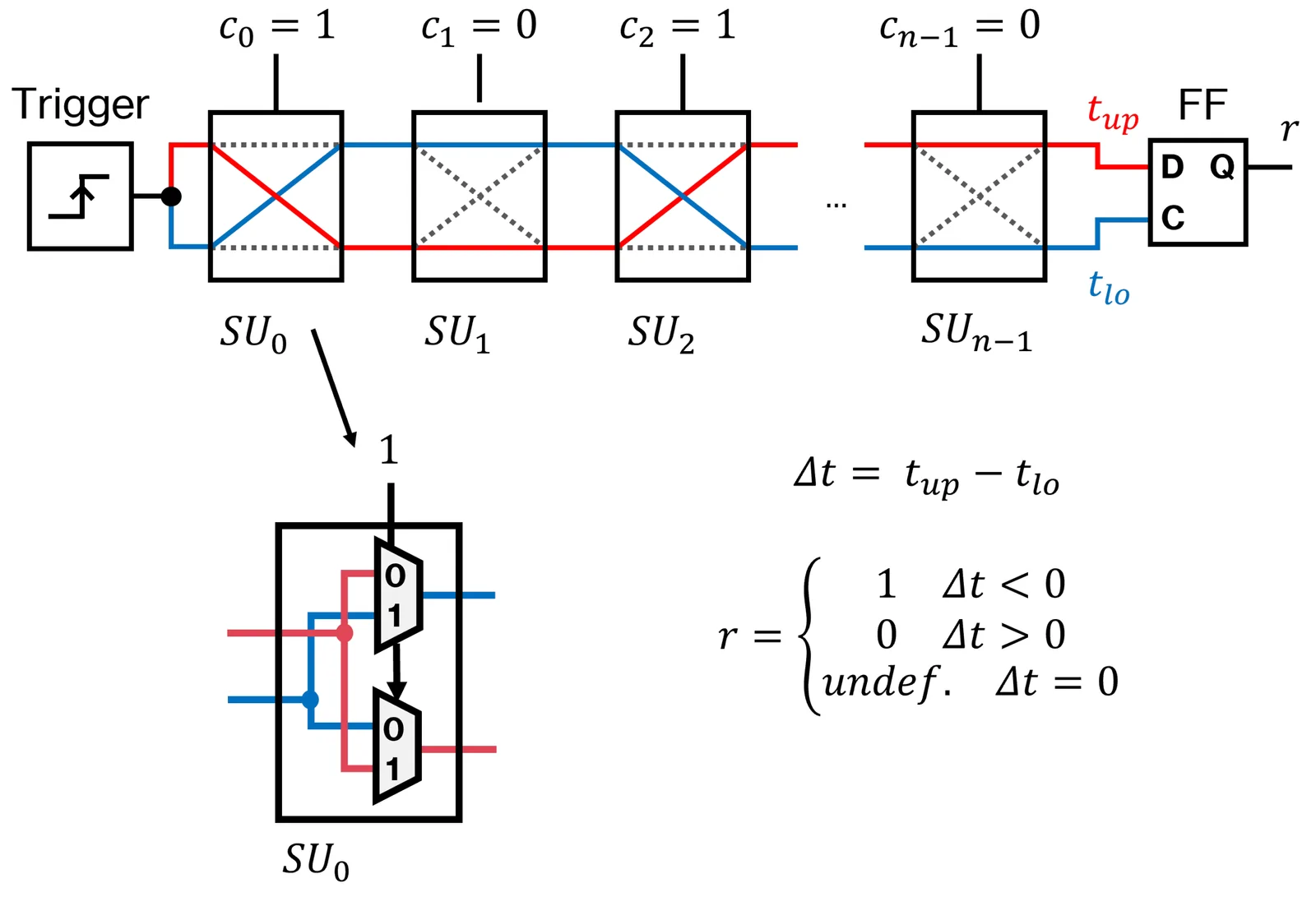
Structure of a basic APUF for generating a single response bit *r*.

The structure of a basic delay-based PUF, referred to as an arbiter PUF (APUF) [30,31], is shown in Figure 1.

In this architecture, a trigger signal is applied at the beginning to activate the PUF. After activation, the signal passes through two parallel paths, where the signal routing can be changed using switching units (SUs). Specifically, the paths are crossed if the corresponding challenge bit *c* is ‘1’ and uncrossed if c=0. As a result, the signal passes through different components depending on the applied challenge [32]. At the end of the PUF, a flip-flop (FF) is used to obtain the response bit [32]. Here, if the upper path is faster than the lower path, the response bit is ‘1’; otherwise, it is ‘0’. Another type of delay-based PUF is the ring oscillator PUF (RO-PUF) [33,34], which is also based on variations in the propagation delays of components within multiple ROs. Delay-based PUFs presented in related work are typically enhanced variants of either APUFs or RO-PUFs.

These differences in signal propagation delays make the response to a specific challenge unique and device-specific. Consequently, a different response is generated when the same PUF design is implemented on a different chip using the same challenge [13,16]. This device-specific response generation is a distinctive feature of PUFs, referred to as the uniqueness characteristic. Another essential attribute is the reliability characteristic, which represents the ability to reconstruct the same response on a specific device when the same challenge is applied [20]. This reproducibility distinguishes delay-based PUFs from true random number generators (TRNGs). The evaluation of both the uniqueness and reliability characteristics is performed with a set of challenges and various devices under different environmental and operational conditions, as presented in detail in [18,20]. Due to these characteristics, PUFs are highly suitable for various security applications, such as PUF-based authentication protocols [35,36,37,38], on-chip key generation primitives [26,39,40], and intellectual property (IP) core obfuscation for know-how protection [41,42]. Furthermore, they can be utilized as TRNGs [43,44], which are typically based on memory-based PUFs. This type of PUF is not considered in this paper.

However, due to various influencing factors ranging from design and operational factors to aging effects [20], both the uniqueness and reliability characteristics are not fully achieved in real PUF implementations. Regarding the uniqueness characteristic, this means that two responses from different devices for the same challenge may not be sufficiently different. In the case of reliability, bit errors can occur when regenerating a response that was previously generated using the same device and challenge. These factors can lead to vulnerabilities or affect the functionality of PUF-based applications, as discussed in detail in the following subsections.

### 2.2. Impact of Operational and Environmental Factors on PUF Reliability

The sensitivity of PUFs to variations in supply voltage (V) and ambient temperature (T) is highlighted in Table 1. Here, it can be observed that the designs cannot achieve an average reliability $Q_{rel}$ of 100%. This is due to the generation of response bits from microscopic differences in component delays, which are affected by environmental and operational conditions, making 100% reliability infeasible over the entire lifecycle of the chip.

The related works presented in Table 1 clearly demonstrate that reliability can be significantly reduced by variations in supply voltage or ambient temperature. The 2-4 DAPUF design implemented in our previous work [18] shows comparatively robust performance, with a difference of only 1.15% between the maximum ($Q_{rel}^{max}$) and minimum ($Q_{rel}^{min}$) reliability values under temperature variations. In contrast, the difference between the minimum and maximum reliability of the Anderson PUF [48] is 30.22%, indicating a substantial reduction in reliability. For a response length of 128 bits, a reduction of 30.22% corresponds to an average of approximately 38.68 bit errors per response. Additionally, aging effects may further decrease reliability. Depending on the PUF-based application, such a reduction in reliability may impair its functionality or introduce security vulnerabilities.

As previously discussed, PUFs can be combined with error-correction mechanisms to correct bit errors in the responses and enhance reliability. To this end, Bose–Chaudhuri–Hocquenghem (BCH) codes are used in [21] to improve PUF reliability from 94.50% to 100% under the same conditions. The reliability of the multipurpose PUF presented in [24] is improved to 100% using BCH and Reed–Solomon codes. In [25], Reed–Muller codes are used in combination with PUFs, while LDPC codes are used in [22]. In [26], the area and power overheads of different ECCs used to improve PUF reliability are investigated. However, the presented works do not investigate the error-correction performance and therefore the resulting reliability of the implemented PUFs under different operating conditions.

In particular, low-rate ECCs may significantly improve reliability and provide high error-correction capability [20], even in the case of aging effects [27] or targeted electromagnetic fault injection attacks [50]. Therefore, this work focuses on the potential security vulnerabilities of PUFs that employ low-rate ECCs to improve their reliability.

## 3. Preliminaries

In this section, preliminary considerations for the security analysis are presented. First, the structure and components of our system model, a PUF-based security application, are described in detail. To this end, this section presents the model for simulating PUF soft responses and additional assumptions for the simulated PUF. Then, for the security analysis, two threat models representing typical attack scenarios against PUF-based applications are discussed. In both attack scenarios, the aim of the attacker is to reconstruct the PUF response of the attacked device. This response serves as the secret cryptographic key in our system model.

### 3.1. System Model

**Figure 2:**
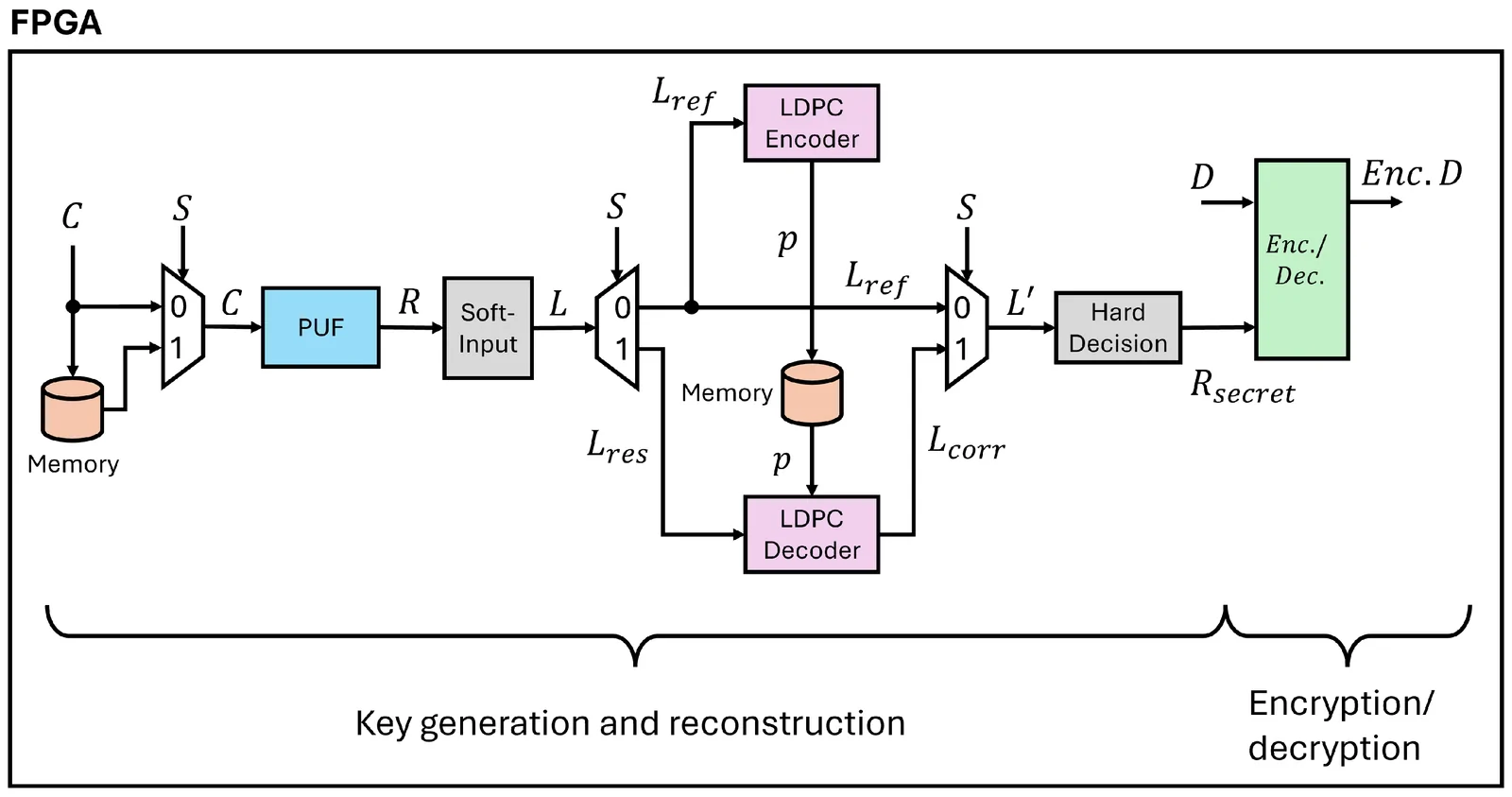
Architecture of the proposed system model for utilizing PUFs as an on-chip cryptographic key generator.

An overview of the proposed system model is presented in Figure 2. In this model, it is assumed that the architecture is implemented on a conventional FPGA, where the bitstream is not encrypted and no additional security measures are used. As a result, an attacker may gain physical access to the FPGA chip and the bitstream stored in external non-volatile memory. In addition, it is assumed that the PUF-integrated device operates under normal operating conditions.

In this system model, the PUF is used to generate cryptographic keys (responses) to encrypt and decrypt sensitive or process-related data *D*. Here, the system generates the key on demand using a challenge *C* and does not use or store fixed keys in non-volatile or on-chip memory. However, to reconstruct the key used for encryption, the PUF is combined with an LDPC encoder and decoder block to correct unavoidable bit errors in the responses and thus avoid incorrect key reconstruction. Otherwise, the encrypted data cannot be decrypted. This proposed PUF-based application significantly decreases the risk of an attacker reconstructing the key stored in memory in conventional symmetric-key approaches through side-channel attacks [3,51,52,53], fault injection [54,55,56], or reverse-engineering attacks [3]. Therefore, the PUF, in combination with an ECC, must ensure 100% reliability. This is typically achieved using low-rate ECCs, specifically an LDPC code in this case. This system model thus represents a PUF-based application with high reliability requirements.

To improve clarity, the encryption/decryption procedure can be controlled by using a control signal *S*, where S=0 is used for encryption while S=1 is used for decryption.

For encryption (S=0), the implemented PUF is used to generate multiple responses *R*. These responses are then used to generate the corresponding soft input *L*. This procedure is described in detail in the following subsection. Next, the soft input *L* is encoded using a soft LDPC encoder to calculate the parity information *p* required for decoding. Subsequently, a hard-decision decoding is performed on the soft input to determine the secret response $R_{secret}$. This decoded response $R_{\mathrm{secret}}$ therefore represents the cryptographic key in this system model. After the encryption process, the challenge *C* and parity information *p* are stored in memory.

For decryption (S=1), the previously stored challenge *C* and parity information *p* are used to reconstruct the secret key used for encryption. To this end, the challenge is used to generate multiple responses, and the corresponding soft input *L* is calculated similarly to the encryption process. Due to the non-ideal characteristics of PUFs and the sensitivity to environmental and operational factors, as presented in detail in [20], the generated soft input *L* may contain bit errors. To correct these errors, the soft input $L_{res}$ (where $L_{res}=L$) is decoded by using the parity information *p* and the LDPC decoder. Finally, the resulting corrected soft input $L_{corr}$ is used to obtain the secret response $R_{secret}$ via hard-decision decoding. Consequently, the decoding process either successfully reconstructs the cryptographic key or fails due to a high error rate, leaving the encrypted data permanently inaccessible.

In typical applications with ECCs, such as data transmission or reliable data storage, the parity information is not considered secret and can therefore be stored in external memory or transferred together with the data over a network. However, in the presented system model, the parity information is generated from the secret response and is used for the reconstruction of the secret key. Therefore, it may leak information about the secret response. In this case, potential information leakage from the parity information should be investigated.

The proposed PUF-based key generation model in Figure 2 provides several advantages compared to the local storage of a cryptographic key in conventional approaches, provided that the implemented PUF demonstrates sufficient reliability and uniqueness. First, due to the uniqueness of the PUF, an attacker cannot reconstruct the secret response solely by gaining access to the challenge *C*. Additionally, the adversary would require detailed information regarding the internal structure and physical placement of the PUF [20], which are not directly disclosed. Second, the parity information alone does not provide sufficient information for an attacker to reconstruct the secret response since the response generated by a challenge *C* on an unauthorized device differs significantly from that of the victim device when using a PUF with enhanced uniqueness.

In this system model, we used a statistical model for PUF soft response generation, as initially proposed in [20]. To this end, we present a refined and extended version of the model, which directly generates the reference as well as the second soft response for reconstruction ($L_{ref}$, $L_{res}$). The model uses statistical characteristics of bit errors and bit stability in the responses obtained from an implemented PUF design, enabling the generation of more realistic soft responses. Therefore, it also allows simulating different scenarios by varying these parameters to evaluate various operating conditions, as demonstrated in previous work [20]. The statistical characteristics of bit errors and bit stability are based on our previously implemented and comprehensively analyzed 2-4 DAPUF [18] as presented in [20].

In the following, both the LDPC codes and the model for soft PUF response generation employed in this security analysis are presented. Furthermore, additional model assumptions are discussed.

#### 3.1.1. LDPC Codes

Table 2 lists the LDPC codes used in this security analysis, along with the corresponding codeword length *N*, soft-information length *K*, and parity information length *P*. For clarity, these LDPC codes are denoted by $W_{R}^{N}$.

These codes are combined with the simulated 2-4 DAPUF design and evaluated under varying ambient temperatures (0 °C, 10 °C, 35 °C, and 50 °C), representing normal operating conditions. In particular, in our comprehensive reliability analysis, the $W_{1/2}^{576}$ code demonstrated superior error-correction performance for all tested scenarios [20]. Based on the reliability analysis methodology for soft responses presented in [20], additional measurements were conducted for the remaining codes listed in Table 2. Therefore, all codes achieved a PUF reliability of 100% under normal operating conditions, which is why they are used in this security analysis.

Furthermore, in the comprehensive reliability analysis, the error-correction performance of the LDPC codes was evaluated and compared for both soft inputs and limited-precision (LP) soft inputs [20]. Specifically, for soft inputs, the PUF is activated multiple times to estimate the probabilities $P(r_{i}=0)$ and $P(r_{i}=1)$ for each response bit $r_{i}$. These probabilities are then used to calculate the log-likelihood ratio (LLR) for each bit according to Equation (1), as in our system model. In the case of P(r=0)=100% or P(r=1)=100%, which represents maximum stability, a maximum absolute LLR value $L_{max}$ of 7.75 is used. As a result, each soft input is represented by an LLR value in the range [−7.75,7.75] and therefore contains crucial information about bit stability and potential errors obtained from multiple PUF activations.

(1) $$L\left(r_{i}\right)=\log\left(\frac{P(r_{i}=0)}{P(r_{i}=1)}\right)$$

For the LP soft inputs, the PUF is activated only once before the LLR values are calculated. This means that each LLR value is either −7.75 or 7.75 for a response bit of 1 or 0, respectively. Therefore, the LP soft inputs can be considered binary inputs from which information about bit errors can be obtained, while information about bit stability is not provided [20]. As a result, the comprehensive reliability analysis in [20] showed that the error-correction performance of the LDPC codes was significantly higher when using soft inputs instead of LP soft inputs due to the additional bit-stability information contained in the soft responses. However, the soft inputs require multiple PUF activations.

In this system model, the simulated 2-4 DAPUF is therefore combined with the codes listed in Table 2. As previously discussed, the selection of the $W_{1/2}^{576}$, $W_{2/3}^{576}$, and $W_{3/4}^{576}$ codes is based on our previous comprehensive reliability analysis [20] in which these codes demonstrated high error-correction performance for the considered PUF responses. The $W_{1/2}^{768}$ and $W_{1/2}^{864}$ codes are additionally considered to extend the investigation to further WiMAX code configurations. In particular, the code pairs $W_{2/3}^{576}$ and $W_{1/2}^{768}$, as well as $W_{3/4}^{576}$ and $W_{1/2}^{864}$, have the same information length *K* while differing in their parity length *P*. This enables an isolated analysis of the security impact associated with the parity length of the corresponding codes. Under the considered temperature variations, all the codes improved the PUF reliability to 100%, demonstrating their suitability for PUF-based applications. Furthermore, a maximum of 25 decoding iterations is used for both input types, which are also considered in the threat models presented in the following subsection.

The quantitative results of this work cannot be directly transferred to other ECC families, such as BCH, Reed–Solomon, polar codes, or to other LDPC configurations. These codes may differ in their parity information and error-correction characteristics, which can affect the probability of a successful attack. In this case, the security impact of other ECC mechanisms should be investigated separately.

#### 3.1.2. PUF Design Characteristics and Proposed Model for Response Generation

**Figure 3:**
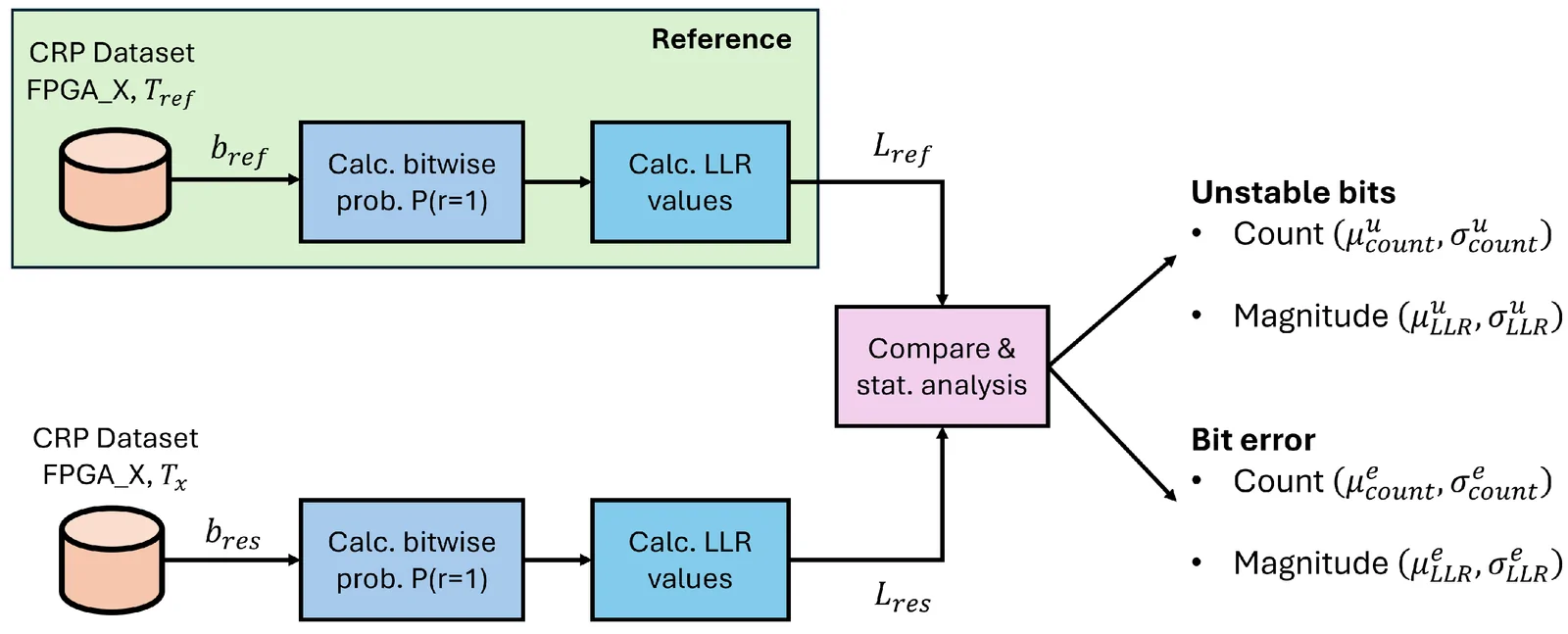
Architecture for the calculation of the statistical parameters used in the proposed PUF response model based on the methodology presented in [20].

The architecture for calculating the statistical parameters for bit stability and bit errors in the soft responses used in the proposed model is shown in Figure 3. As shown in this architecture, the collected CRPs of a PUF design for a specific FPGA (FPGA_X) at a temperature of $T_{ref}$ are used as a reference and compared with those at a second temperature $T_{x}$. For both temperatures, multiple binary responses *b* for a specific challenge are collected, and the LLR values are calculated as previously presented [20]. Then, both the reference soft response $L_{ref}$ and the second soft response $L_{res}$ are compared, and the statistical parameters for bit instability and bit errors are evaluated. For this analysis, it is assumed that the reference response $L_{ref}$ does not contain any bit errors. Here, an unstable bit is defined as a state where a soft response bit does not reach the maximum absolute LLR value of $L_{max}$, while the sign of the reference soft response at the same position remains unchanged [20]. Furthermore, if the LLR value is close to $\pm L_{max}$, it is defined as a highly stable bit, whereas a lower LLR value indicates high instability. As a result, the highest instability for a soft input bit occurs when the absolute LLR value is close to 0, while the highest stability is achieved when the LLR value is the same as that of the reference at this position [20]. However, a bit error in the soft responses occurs when the signs of a soft response bit in both responses at the same position differ [20]. In this case, a weak bit error is defined as an error where the absolute LLR value is close to 0. For higher absolute LLR values with opposite signs, the bit errors are defined as strong bit errors [20].

Consequently, the count parameters correspond to the distributions of the number of unstable bits ($\mu_{count}^{u},\sigma_{count}^{u}$) and bit errors ($\mu_{count}^{e},\sigma_{count}^{e}$), while the magnitude parameters correspond to the distributions of the LLR values of unstable bits ($\mu_{LLR}^{u},\sigma_{LLR}^{u}$) and bit errors ($\mu_{LLR}^{e},\sigma_{LLR}^{e}$). This procedure can be applied to any PUF design, thereby providing essential information regarding bit stability and errors, as comprehensively investigated in [20]. The presented analysis and the corresponding statistical characteristics therefore represent part of the reliability analysis of a PUF design, in which the responses obtained under different temperature conditions for the same challenge are evaluated against a reference response.

The statistical characteristics of the 56-bit 2-4 DAPUF design [18,20] at various ambient temperatures are presented in Table 3. For the evaluation of these statistical characteristics, a reference temperature $T_{ref}$ of 20 °C is used. In this table, it can be observed that the 56-bit 2-4 DAPUF design generates 3.7 unstable bits on average, which corresponds to 6.60% of the total number of response bits across the evaluated temperatures. On average, this instability increases with increasing temperature, which can be observed from the decreasing $\mu_{LLR}^{u}$ value. In contrast, this design generates 1.6 bit errors on average for the evaluated temperatures, which corresponds to 2.86% of the total number of response bits. The lowest number of bit errors is achieved at 10 °C, while the highest number of bit errors of approximately 1.817 bits is achieved at 50 °C. Furthermore, these bit errors are much more pronounced at 0 °C and 50 °C compared to 10 °C and 35 °C due to the higher values of $\mu_{LLR}^{e}$ and $\sigma_{LLR}^{e}$, resulting in statistically higher LLR values.

**Figure 4:**
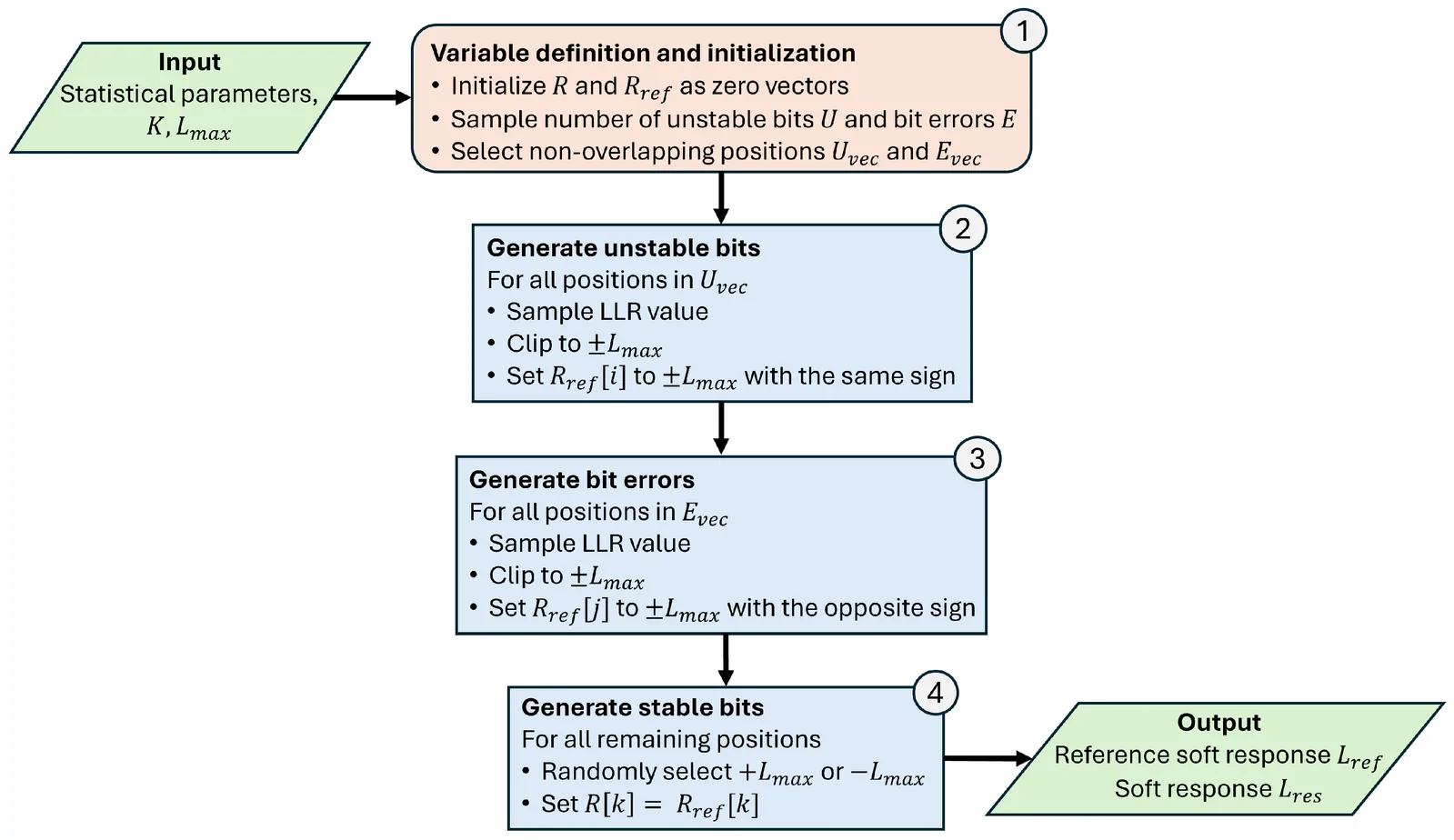
Flowchart of the processing steps of the refined and optimized statistical PUF response generation model.

Figure 4 illustrates the flowchart of the processing steps of the refined and optimized statistical PUF response model [20]. The model consists of four main processing steps: variable definition and initialization, generation of unstable bits, generation of bit errors, and generation of stable bits. The corresponding detailed pseudocode is presented in Algorithm 1. In addition to the statistical parameters, the response length *K* and the maximum LLR value $L_{max}$ are required to generate simulated soft responses. Here, a simulated response corresponds to the soft input obtained from several binary responses of a PUF design and represents *L* in our system model in Figure 2.

In the first step (lines 3–8), two zero vectors, *R* and $R_{ref}$, representing the soft responses and the reference response, respectively, are generated. In addition, two random variables, *U* and *E*, representing the number of unstable bits and bit errors, respectively, are generated. Different distributions can be used for this purpose. In this work, both the number of unstable bits and the number of bit errors are assumed to follow normal distributions, with their parameters derived from the CRPs of the implemented 2-4 DAPUF. In this case, *U* represents the number of unstable bits and is generated by using a Gaussian distribution with $\mu_{count}^{u}$ and $\sigma_{count}^{u}$ ($N(\mu_{count}^{u},\sigma_{count}^{u\;2})$), while *E* represents the number of bit errors in the soft responses, using $\mu_{count}^{e}$ and $\sigma_{count}^{e}$ ($N(\mu_{count}^{e},\sigma_{count}^{e\;2})$). Then, two additional vectors, $U_{vec}$ and $E_{vec}$, are defined. Here, vector $U_{vec}$ of length *U* contains randomly chosen indices of vector *R* and represents the bit positions of unstable bits in the generated soft response. Similarly, $E_{vec}$, with a length of *E*, contains randomly chosen indices of *R*, representing the positions of bit errors. For both vectors, $U_{vec}$ and $E_{vec}$, each index is chosen only once, meaning that an index in $U_{vec}$ cannot be in $E_{vec}$ and vice versa [20]. After the first processing step, the vectors for the soft responses, the number, and the bit positions of unstable bits and bit errors are defined.

In the second processing step (lines 9–23), unstable soft response bits are simulated for all elements of $U_{vec}$. For this purpose, random samples (tmpVar) are generated using the corresponding parameters $\mu_{LLR}^{u}$, $\sigma_{LLR}^{u}$, representing the LLR values of the unstable soft inputs. As with the number of unstable bits and bit errors, different distributions can be used depending on the statistical model of the LLR values. However, in this work, the LLR values are modeled using a Gaussian distribution. Next, it is checked whether a generated sample exceeds the maximum absolute LLR value $L_{max}$ (lines 11–15). In this case, the sample is clipped to either $-L_{max}$ or $+L_{max}$ based on the sign of the random sample and stored in R[i]. Otherwise, the generated sample is stored unchanged in *R* at the corresponding index *i*. To simulate the reference soft response ($R_{ref}$) at position *i*, the LLR value is set to either $-L_{max}$ or $+L_{max}$, using the same sign as the generated sample. However, if the generated sample is equal to zero, which represents the highest instability, the corresponding reference response is randomly set to either $-L_{max}$ or $+L_{max}$. Thus, the signs of both soft responses are identical. The LLR value stored in *R* lies within the range $\left[-L_{max},+L_{max}\right]$, whereas the error-free reference response is either $-L_{max}$ or $+L_{max}$.
**Algorithm 1** Improved statistical model for the simulation of soft responses based on the model proposed in [20].  1:**Input:** *K*, $\mu_{count}^{u}$, $\sigma_{count}^{u}$, $\mu_{LLR}^{u}$, $\sigma_{LLR}^{u}$, $\mu_{count}^{e}$, $\sigma_{count}^{e}$, $\mu_{LLR}^{e}$, $\sigma_{LLR}^{e}$, $L_{max}$  2:**Output:** $L_{ref}$, $L_{res}$  3:R← create zero vector of length *K*                                                                                     ▷Variable initialization  4:$R_{ref}\leftarrow$ create zero vector of length *K*  5:U← random sample using $\mu_{count}^{u},\sigma_{count}^{u\;2}$                                 ▷ Sample number of unstable bits and bit errors  6:E← random sample using $\mu_{count}^{e},\sigma_{count}^{e\;2}$  7:$U_{\mathrm{vec}}\leftarrow$ select *U* random indices from *R*                           ▷ Determine positions of unstable bits and bit errors  8:$E_{\mathrm{vec}}\leftarrow$ select *E* random indices from *R*  9:**for** each index *i* in $U_{\mathrm{vec}}$ **do**                                                                                                   ▷ Simulate unstable bits10:    tmpVar← random sample using $\mu_{LLR}^{u},\sigma_{LLR}^{u\;2}$                                                                        ▷ Sample LLR value11:    **if** $\mathrm{abs}\left(tmpVar\right)>L_{max}$ **then**                                                                              ▷ Generate simulated response12:        $R\left[i\right]\leftarrow \mathrm{sign}\left(tmpVar\right)\cdot L_{max}$13:    **else**14:        R[i]←tmpVar15:    **end if**16:    **if** R[i]>0 **then**                                                                                                      ▷ Generate reference response17:        $R_{ref}\left[i\right]\leftarrow L_{\max}$18:    **else if** R[i]<0 **then**19:        $R_{ref}\left[i\right]\leftarrow -L_{\max}$20:    **else**21:        $R_{ref}\left[i\right]\leftarrow \mathrm{rand}\left\{-1,+1\right\}\cdot L_{max}$22:    **end if**23:**end for**24:**for** each index *j* in $E_{\mathrm{vec}}$ **do**                                                                                                     ▷ Simulate bit errors25:    tmpVar← random sample using $\mu_{LLR}^{e},\sigma_{LLR}^{e\;2}$                                                                   ▷ Sample LLR value26:    **if** $\mathrm{abs}\left(tmpVar\right)>L_{max}$ **then**                                                                            ▷ Generate simulated response27:        $R\left[j\right]\leftarrow \mathrm{sign}\left(tmpVar\right)\cdot L_{max}$28:    **else**29:        R[j]←tmpVar30:    **end if**31:    **if** R[j]>0 **then**                                                                                                  ▷ Generate reference response32:        $R_{ref}\left[j\right]\leftarrow -L_{\max}$33:    **else if** R[j]<0 **then**34:        $R_{ref}\left[j\right]\leftarrow L_{\max}$35:    **else**36:        $R_{ref}\left[j\right]\leftarrow \mathrm{rand}\left\{-1,+1\right\}\cdot L_{max}$37:    **end if**38:**end for**39:**for** each remaining index *k* in *R* not in $\left(U_{\mathrm{vec}}\cup E_{\mathrm{vec}}\right)$ **do**                                          ▷ Generate stable response40:    $R\left[k\right]\leftarrow \mathrm{rand}\left\{-1,+1\right\}\cdot L_{max}$41:    $R_{ref}\left[k\right]\leftarrow R\left[k\right]$42:**end for**43:$L_{ref}\leftarrow R_{ref}$44:$L_{res}\leftarrow R$45:**return** $L_{ref}$, $L_{res}$

Similarly, in the next processing step (lines 24–38), bit errors in the soft response are simulated for all elements of $E_{vec}$. For this purpose, a random sample (tmpVar) is generated using the parameters $\mu_{LLR}^{e}$ and $\sigma_{LLR}^{e}$, representing the LLR value at a bit-error position. Next, it is checked whether the LLR value exceeds the maximum absolute LLR value $L_{max}$. If so, the value is clipped to either $-L_{max}$ or $+L_{max}$ depending on the sign of the random sample and stored in R[j], similarly to the unstable soft response bits. This resulting value represents the strongest possible error. Otherwise, the generated sample is stored unchanged in *R* at position *j*. The reference response $R_{ref}$ is generated similarly to the unstable soft responses. The main difference is that the reference response position is set to either $-L_{max}$ or $+L_{max}$, with the sign opposite to that of the LLR value in R[j]. Thus, if the generated LLR value for a bit error is positive, the corresponding reference soft response bit is set to $-L_{max}$, whereas for a negative LLR value, it is set to $+L_{max}$. If the sample is equal to zero, representing the weakest bit error, the corresponding reference response is randomly set to either $-L_{max}$ or $+L_{max}$, similarly to the unstable bits.

In the final processing step (lines 39–42), highly stable soft responses are simulated. In this case, the same LLR value, either $-L_{max}$ or $+L_{max}$, is used for both responses *R* and $R_{ref}$ for the remaining bit positions. Finally, the generated responses *R* and $R_{ref}$ are assigned to the corresponding outputs $L_{res}$ and $L_{ref}$, respectively (lines 43-44).

**Figure 5:**
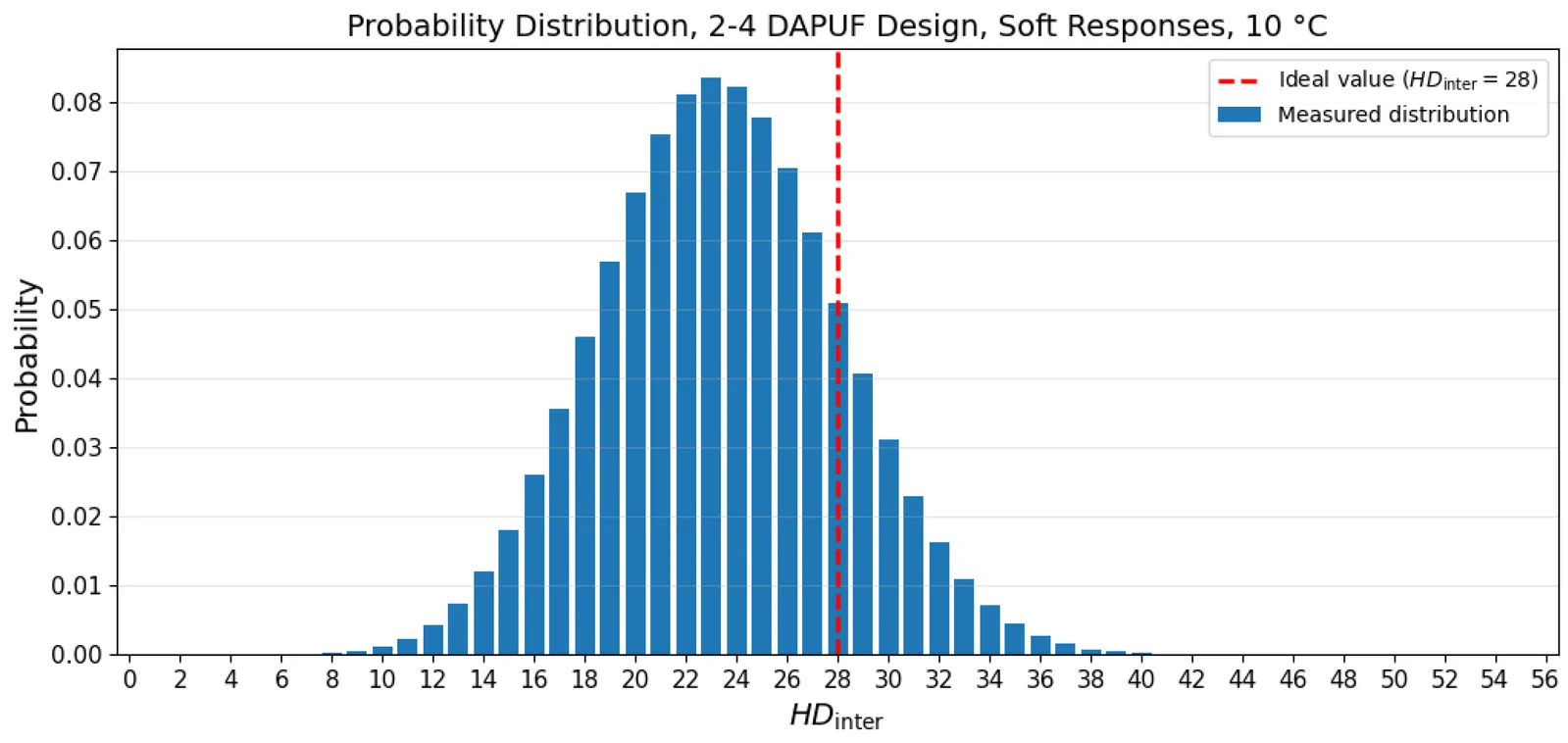
Probability distribution of the inter-Hamming distance of the implemented 2-4 DAPUF used for the security analysis in this work.

Regarding the uniqueness characteristic of the PUF design, the probability distribution of the inter-Hamming distance ($HD_{inter}$) of the 2-4 DAPUF design, representing the number of bit differences in the soft responses for the same challenge across multiple FPGAs and different CRPs, is investigated, as illustrated at a temperature of 10 °C in Figure 5. Here, it can be observed that the 2-4 DAPUF design demonstrates good uniqueness characteristics. On average, the responses generated by this PUF design differ in approximately 23 bits (41.07%) when implemented on different FPGAs using the same challenge. However, lower numbers of bit differences can also occur. For the different temperatures of 0 °C, 10 °C, 35 °C, and 50 °C, the $HD_{inter}$ distribution is evaluated separately for each temperature considered in our threat models.

#### 3.1.3. Additional Model Assumptions for Security Analysis

For the security analysis, soft-information lengths *K* of 288, 384, and 432 bits are used for the considered LDPC codes listed in Table 2. To extend the soft-response length to the required *K* values for the different LDPC code configurations while preserving the same statistical characteristics, the individual 56-bit DAPUF blocks are assumed to be statistically independent. This assumption is motivated by the structure of APUFs and the implemented 2-4 DAPUF, as discussed in the following.

As illustrated in Figure 1, a basic APUF block generates a single response bit by comparing the propagation delays of two signal paths. Typically, to generate multiple response bits, the same APUF block structure is implemented multiple times in parallel. In related work, such individual APUF instances are considered independent PUF structures [58]. Furthermore, the response bits of APUFs can be considered statistically independent for challenges with a sufficiently large HD [59]. This condition is typically satisfied for randomly selected challenges, as is the case in this work.

The implemented 2-4 DAPUF follows a similar principle but consists of four signal paths, as presented in [18,29]. Each 2-4 DAPUF instance is implemented using separate switching units, signal paths, and FFs. Similar to APUFs, multiple individual 2-4 DAPUF instances are implemented on the FPGA to generate multiple response bits and form the 56-bit 2-4 DAPUF. To extend the soft-information length to the required *K*, several 56-bit DAPUF blocks are concatenated. Based on this structure, these 56-bit DAPUF blocks are considered statistically independent under the assumptions of the proposed model.

For the count parameters describing the number of unstable bits and bit errors, the mean and variance are assumed to scale linearly with the soft-response length. For this purpose, the mean is scaled by a factor of *k*, while the standard deviation is scaled by a factor of $\sqrt{k}$ according to Equation (2). The LLR magnitude parameters characterize the soft-information distribution of individual bits and are therefore assumed to be independent of the overall response length.

(2) $$\begin{array}{cc} k & =\frac{K}{56}\\ \mu_{count}^{u/e} & \to k\cdot \mu_{count}^{u/e}\\ \sigma_{count}^{u/e} & \to \sqrt{k}\cdot \sigma_{count}^{u/e} \end{array}$$

To determine the $HD_{inter}$ distribution for a response length of *K* bits at different temperatures, the same assumptions of statistical independence and identical statistical characteristics are applied to the individual PUF blocks. Since the total $HD_{inter}$ for a soft-information length of *K* is given by the sum of the $HD_{inter}$ of the individual PUF blocks, its discrete probability distribution can be obtained by convolution. Accordingly, the distribution for complete *K*-bit response segments is obtained by repeated self-convolution of the measured 56-bit distribution (Figure 5). For response lengths that are not integer multiples of 56 bits, the $HD_{inter}$ distribution of the remaining response bits is evaluated separately. For this purpose, the first *Y* bits (Y=Kmod56) of the measured 56-bit responses are extracted and evaluated separately to obtain the corresponding *Y*-bit $HD_{inter}$ distribution, which then models the remaining *Y*-bit response segment. For example, for K=288 bits, the resulting $HD_{inter}$ distribution is obtained by performing a fivefold convolution of the 56-bit distribution and subsequently convolving the result with the separately determined 8-bit distribution. When implementing a 288-bit PUF using the 2-4 DAPUF structure, the same assumption would apply, resulting in five 56-bit 2-4 DAPUF blocks and two additional 2-4 DAPUF instances for the remaining 8 bits.

These assumptions provide a statistical model for generating soft responses of length *K* at different temperatures that preserve the relevant statistical characteristics of the measured 56-bit 2-4 DAPUF under the stated independence and scaling assumptions. In addition, the approximate probability distribution of the soft responses of length *K* is derived from the corresponding $HD_{inter}$ distribution measured for the implemented PUF design at different temperatures (*T*) and is denoted by $P_{K}^{T}$.

### 3.2. Threat Models

In this subsection, the two threat models considered in this paper are presented. As previously discussed, these threat models represent typical attack vectors against PUFs and PUF-based applications. In both threat models, it is assumed that an attacker attempts to reconstruct the response obtained on the victim device used as a cryptographic key $R_{secret}$ in the system model shown in Figure 2. In addition, this subsection summarizes all assumptions considered in the security analysis.

#### 3.2.1. Device-Cloning Attack

**Figure 6:**
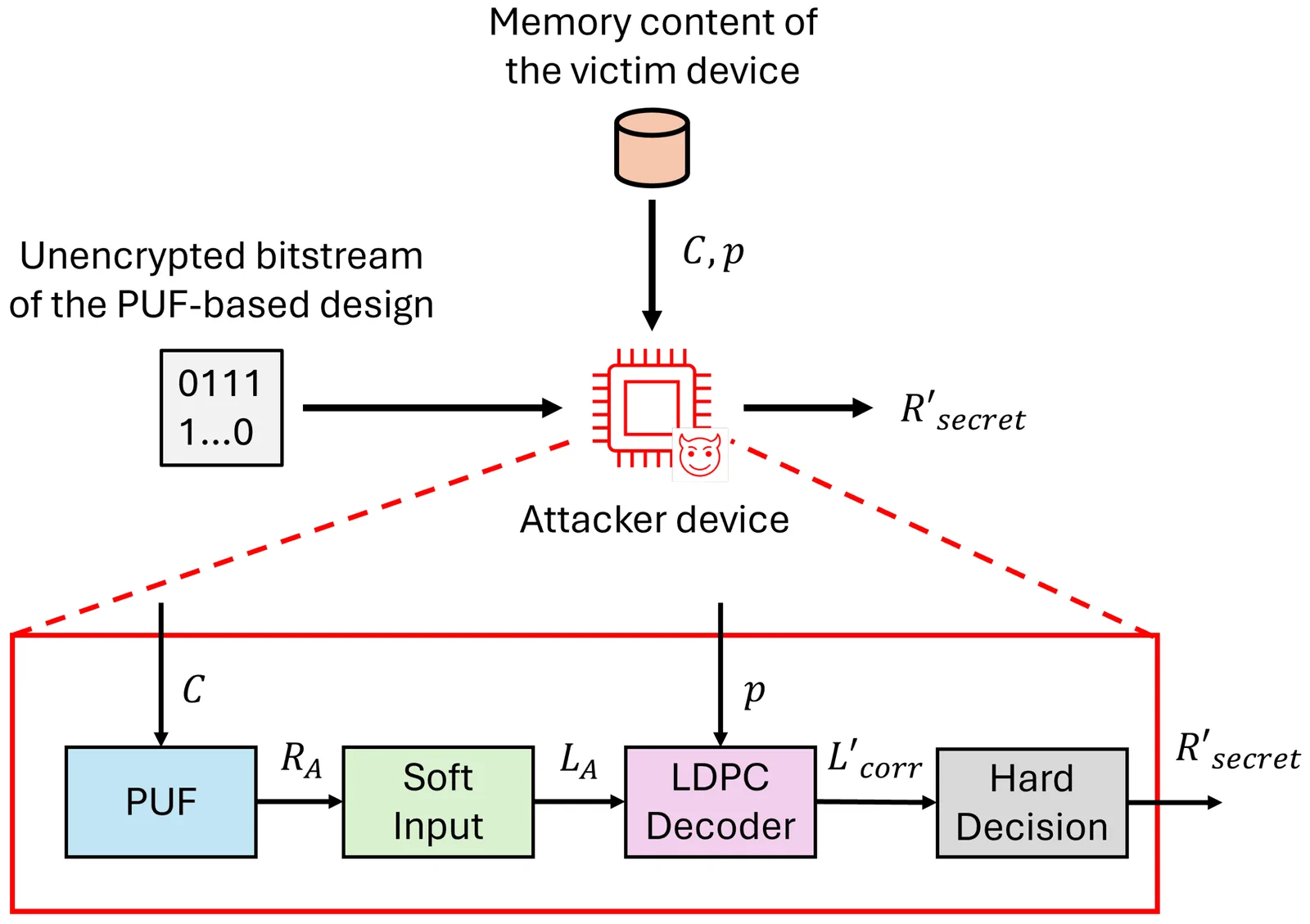
Structure of the first threat model proposed for the security analysis, representing a cloning attack on a PUF-based device to reconstruct the secret response on the attacker’s device.

An overview of the first threat model is shown in Figure 6. In this threat model, an attacker may perform non-invasive attacks, such as side-channel or fault-injection attacks, to gain access to the unencrypted hardware configuration bitstream of the entire design implemented on the victim device. In addition, the attacker can obtain the challenge *C* and the parity information *p*, which are generated and stored after the encryption process of the application (Figure 2).

During a cloning attack, the attacker implements the hardware configuration on a second FPGA belonging to the same device family as the victim device. In this case, the entire design—including all components, the implemented PUF, and their placement and routing—is cloned onto the attacker’s device. Consequently, the attacker can apply the challenge *C* obtained from the memory of the victim device to generate a response using the cloned PUF. Therefore, it can be assumed that the HD between the soft response *L* of the victim device and the soft response $L_{A}$ of the attacker device follows the $HD_{inter}$ distribution shown in Figure 5. Similarly, at a given ambient temperature, the soft responses obtained from the attacker’s device and the victim device exhibit similar stability and bit-error characteristics (Table 3), as both FPGAs belong to the same device family. The reliability of both implementations is improved to 100% using the considered LDPC codes (Table 2).

As a result, in this threat scenario, the attacker can successfully reconstruct the key if the generated soft response does not exhibit excessive bit differences. This enables successful error correction of the victim device’s secret soft response ($L_{corr}^{\prime }=LDPC\_Decode\left(L_{A},p\right)$, where $L_{corr}^{\prime }=L_{corr}$) and the subsequent recovery of the secret key $R_{secret}$ using hard-decision decoding. However, if the two soft responses differ significantly and the LDPC code is unable to correct these bit errors, the attacker cannot reconstruct the response.

In general, ECCs with a low code rate exhibit improved error-correction capability compared to codes with a high code rate. In particular, soft LDPC decoders typically demonstrate superior performance compared to binary decoders due to the use of additional soft information regarding bit-stability and error characteristics. This threat model, therefore, represents a crucial trade-off in hardware security between the necessity of ECCs in PUF-based applications, as discussed in Section 2.2, and the uniqueness characteristic of the PUF, which is represented by the $HD_{inter}$ probability distribution $P_{K}^{T}$.

The considered cloning attack, therefore, represents a strong attacker model, in which the attacker has gained access to the unencrypted hardware configuration bitstream as well as the stored challenge *C* and parity information *p*. Consequently, the resulting attack success probabilities depend on the corresponding assumptions and model parameters in this attack scenario. For example, this security risk can be significantly reduced by using bitstream encryption since the encrypted bitstream cannot be directly used and implemented on the attacker’s device without the corresponding decryption key. In real-world scenarios, the considered assumptions may still apply if such protection mechanisms are not enabled. In addition, bitstream encryption is supported by various modern FPGA families. Furthermore, access to the challenge *C* and parity information *p* may also be obtained through other attack vectors instead of directly reading the memory content. An example of such an attack is a man-in-the-middle attack, in which an attacker intercepts data during data transmission between two nodes. In particular, in some PUF-based authentication protocols, the challenge and parity/helper information may be transmitted over a network to reconstruct the secret response for authentication [60,61,62].

#### 3.2.2. Reverse-Engineering Attack

**Figure 7:**
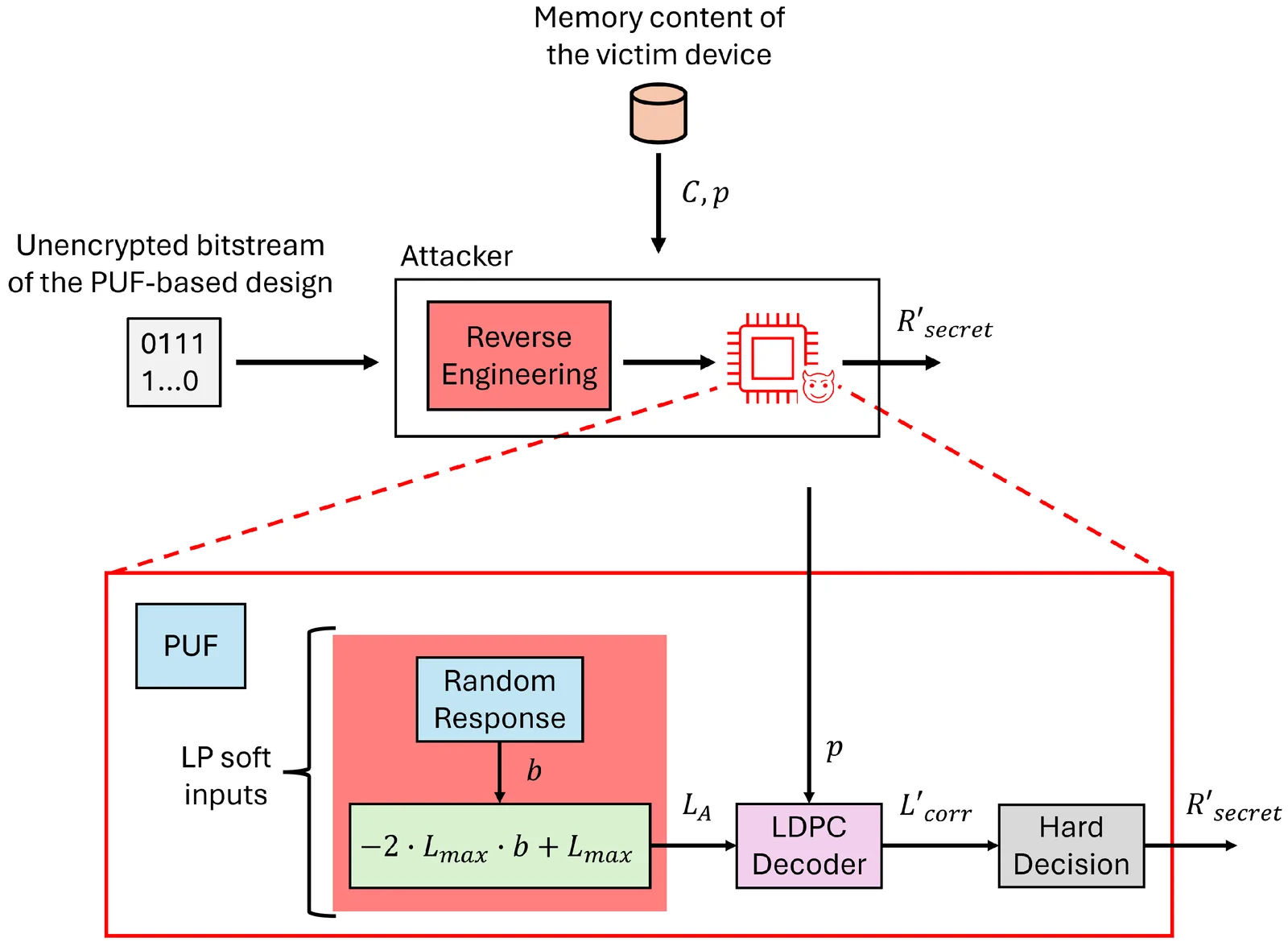
Structure of the second threat model proposed for the security analysis, representing a reverse-engineering attack to reconstruct the secret response from a randomly generated response using LP soft-input decoding.

The structure of the second threat model is shown in Figure 7. In this threat model, it is also assumed that the attacker gains access to the hardware configuration, the challenge *C*, and the parity information *p*, as in the first attack scenario. Unlike in the first threat model, the attacker implements the design on an FPGA from a different device family and with a different architecture than the victim’s device. As a result, the attacker cannot reproduce the victim’s PUF behavior since the placement and routing constraints are specific to the FPGA device family. Instead, the attacker reverse-engineers and extends the design to generate random responses and subsequently calculate LP soft inputs (Figure 7). As a result, the responses generated by the attacker are generally random and contain limited or no information about the bit stability or bit-error characteristics of the victim’s PUF. After generating a random response and converting it into LP soft inputs, the attacker uses the soft LDPC decoder in combination with the parity information *p* to correct bit errors and thereby reconstruct the original response.

As a result, a successful recovery of the original response ($R_{secret}$) in this threat model would demonstrate that the original response can be reconstructed from attacker-generated responses with limited or no information about the bit errors or bit stability of the victim’s PUF, using the parity information and an LDPC code. This would represent a worst-case scenario and a significant vulnerability for the application. However, from a theoretical perspective, the use of LDPC codes with very low code rates may increase the probability of a successful reconstruction, as the additional parity information generally provides a higher error-correction capability. For example, for an ECC with a code rate of 1/4, 25% of the codeword consists of information bits, while the remaining 75% consists of parity bits.

To further investigate the reconstruction of the secret response ($R_{secret}$) under different statistical characteristics of the attacker-generated responses, two cases are considered in this attack scenario. First, the bit differences between the two responses are assumed to be statistically independent, with different *q* values representing the probability that a bit of the attacker-generated response differs from the corresponding bit of the secret response. Accordingly, *q* also represents the expected normalized $HD_{inter}$ between the two responses $R_{secret}$ and $R_{secret}^{\prime }$. Second, the influence of correlations (ρ) between individual bit differences is investigated while keeping q=0.50 constant. For example, such correlations may occur when structural or routing characteristics of a PUF design affect multiple response bits. As a result, bit differences at these positions may not occur independently but may instead be statistically correlated.

The investigated *q* and ρ values are used as sensitivity parameters in the model-based reverse-engineering attack scenario and therefore do not represent experimentally measured characteristics of the implemented 2-4 DAPUF.

Table 4 provides an overview of the statistical models and probability distributions used throughout the security analysis.

## 4. Model-Based Security Evaluation and Discussion

In this section, the model-based results of the security analysis for both threat models are presented. To this end, an algebraic analysis of the information leakage from the stored parity information is first conducted to provide an initial assessment of potential security risks, as the parity information is obtained by the attacker in both attack scenarios. Then, the probability $P_{success}$ that an attacker can successfully reconstruct the secret response $R_{secret}$ for the considered LDPC code configurations listed in Table 2 is evaluated. Here, it should be noted that the presented probabilities $P_{success}$ are derived from the statistical PUF model and the stated model assumptions described in Section 3. These results have not yet been validated through complete hardware attack experiments. However, such hardware validation is planned as part of our future work and is discussed in Section 5.

Probability-based analyses are also used in other security applications to investigate system behavior and potential vulnerabilities. For example, probabilistic models have been applied to analyze signal detection and synchronization processes, as well as security-related attack scenarios in quantum key distribution systems [63,64]. In this work, probability-based methods are used to evaluate the considered PUF-based attack scenarios. Specifically, the probability of successfully reconstructing the secret response is determined for both threat models.

### 4.1. Information Leakage Analysis of the Stored Parity Information

The evaluation of information leakage from the stored parity data is an important security aspect, as the parity information *p* is generated from the secret response and can be obtained by an attacker in both attack scenarios. To investigate this aspect, a rank-based analysis is performed for the considered LDPC codes (Table 2). To this end, the rank of the parity mapping is determined to evaluate the number of independent linear constraints on the secret response. Consequently, the number of remaining degrees of freedom and the corresponding number of possible secret responses can be calculated. Similar rank-based investigations of information leakage from helper-data constructions for PUF-based key generation have been presented in previous work [65]. In addition, ref. [66] discusses the parity construction and its information leakage.

For a linear and systematic LDPC encoder, the parity information *p* can be represented as [65,66]

(3) $$p=AR_{secret}\;\left(\mathrm{mod}\;2\right)$$

Here, *A* is the binary parity mapping matrix of dimension P×K, which generates the parity information *p* from the *K*-bit secret response $R_{secret}$. The rank of *A* is then determined over GF(2) to evaluate the number of independent linear constraints on the secret response [65]. The resulting rank is used to calculate the number of remaining degrees of freedom *d*, as defined by

(4) d=K−rank(A)

Consequently, the number of possible secret responses that are consistent with the stored parity information can be calculated as

(5) $$N_{responses}=2^{d}$$

This means that an attacker who has obtained the stored parity information and knows the employed ECC encoding scheme, as assumed in both attack scenarios, can determine the set of possible response candidates that generate the same parity information. During iterative decoding, the decoder uses the received response and its bit reliability information to estimate a corrected response with the aim of obtaining a codeword that satisfies the parity-check equations [67,68]. If the received response is sufficiently similar to the secret response, this additional information may help the decoder identify the correct response among the $2^{d}$ possible response candidates.

Regarding information leakage, a smaller value of *d* indicates that more independent linear constraints on the secret response $R_{secret}$ are provided by the stored parity information, resulting in fewer possible secret response candidates $N_{responses}$. On the other hand, a larger value of *d* corresponds to a larger number of possible response candidates.

The calculated *d* values for the considered WiMAX codes are shown in Table 5. Here, it should be noted that the presented rank-based analysis determines the number of algebraically possible secret responses consistent with the parity information. The actual remaining entropy depends on the statistical distribution of the secret response and may differ for biased or correlated PUF responses [69]. Therefore, this investigation represents an initial assessment of the information leakage from the parity information.

In Table 5, it can be observed that the codes with a code rate of 1/2 exhibit smaller *d* values than the codes with rates of 2/3 and 3/4. The numbers of response candidates consistent with the same parity information are 32, 16, and 256 for the $W_{1/2}^{576}$, $W_{1/2}^{768}$, and $W_{1/2}^{864}$ codes, respectively. For the considered codes, the higher number of parity bits *P* relative to the information length *K* is associated with a higher rank of the parity mapping matrix and, consequently, a larger number of independent linear constraints on the secret response. The $W_{2/3}^{576}$ and $W_{3/4}^{576}$ codes, in particular, result in a larger number of response candidates for the same parity information. As a result, the considered codes with a rate of 1/2 exhibit greater algebraic information leakage than the investigated higher-rate codes.

### 4.2. Security Evaluation of the Device-Cloning Attack

The security evaluation of the device-cloning attack scenario is performed in two steps. Due to the use of soft responses with different bit-stability and bit-error characteristics across different temperatures (Table 3), the error-correction performance of the employed LDPC codes is assessed as a function of the evaluated ambient temperatures of 0 °C, 10 °C, 35 °C, and 50 °C in the first step. To this end, the decoding success rate (DSR) is analyzed, which represents the percentage of successfully corrected soft responses out of a total of 5000 responses.

For example, a DSR of 0.65 (65%) means that 3250 soft responses are fully corrected, while 1,750 responses still contain bit errors after the decoding process. To evaluate the statistical uncertainty of the DSR values, the standard error SE is calculated according to Equation (6), assuming independent decoding trials.

(6) $$SE=\sqrt{\frac{DSR(1-DSR)}{n}}$$

For DSR=0.65 and n=5000, the resulting standard error SE is approximately 0.675 percentage points. Since a total of n=5000 soft responses is evaluated for each investigated number of bit errors $\mu_{count}^{e}$, the maximum standard error is approximately 0.71 percentage points and occurs at a DSR of 0.5 (50%). For this DSR, the corresponding approximate 95% confidence interval is 50% ± 1.39 percentage points. However, this interval only considers the statistical uncertainty due to the limited number of simulated responses.

The DSR is then evaluated for all LDPC codes and temperatures for different fixed numbers of bit errors ($\sigma_{count}^{e}=0$) introduced at random bit positions. The aim of this investigation is to determine the DSR under the given conditions at different temperatures while the number of bit errors is varied from 0 to *K*. This identifies the error-correction limits of the LDPC codes for each ambient temperature condition. In the second step, the scaled probability distribution of the $HD_{inter}$ for *K* response bits is multiplied by the evaluated DSR values of the corresponding code rate for each temperature *T* in order to calculate the overall probability of successful soft-response reconstruction $P_{success}\left(T\right)$ by the attacker.

**Figure 8:**
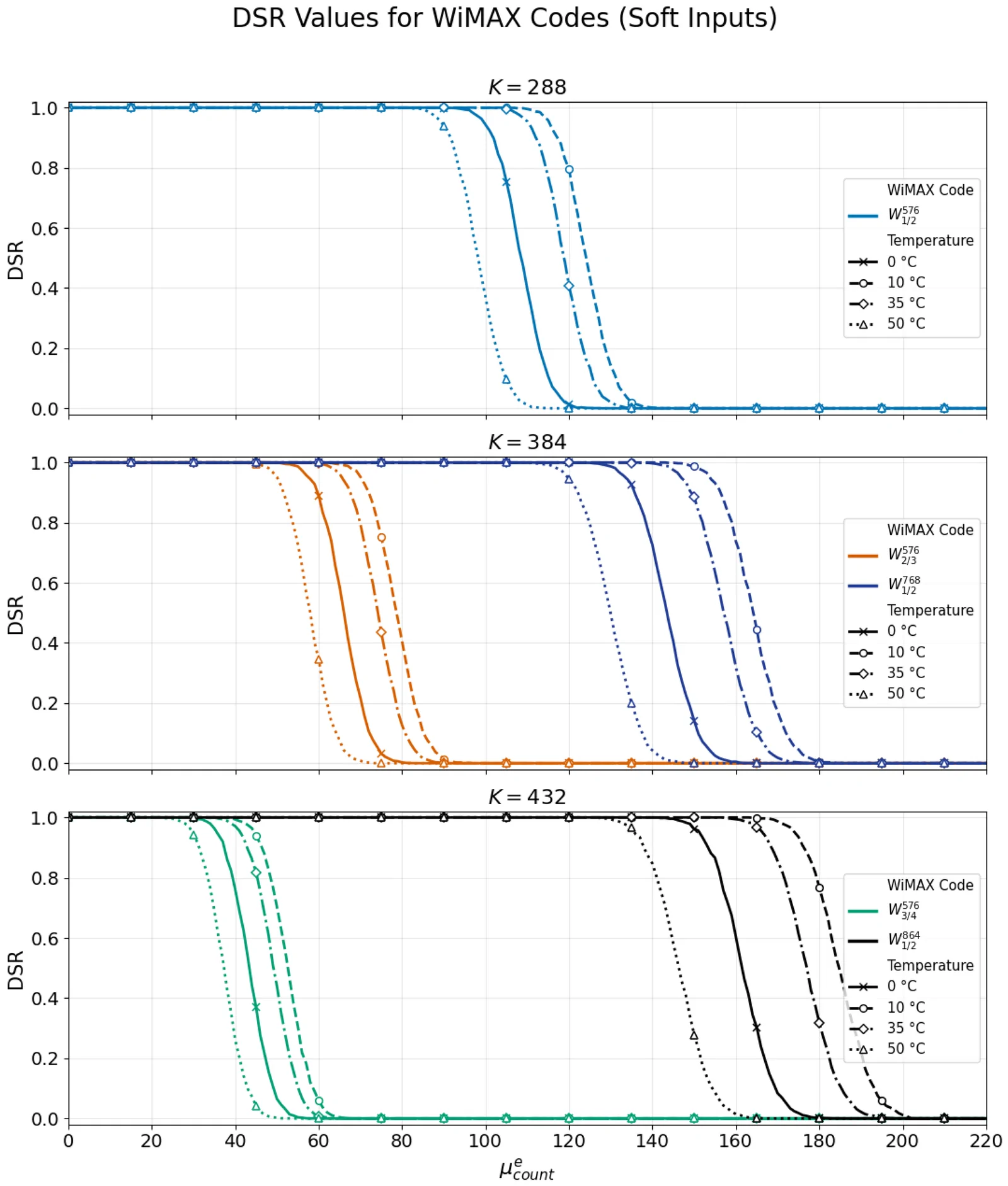
Decoding success rate (DSR) for the considered LDPC codes and ambient temperatures as a function of the number of bit errors $\mu_{count}^{e}$ in the soft responses for the first threat model.

Figure 8 presents the DSR for the considered LDPC codes and temperatures as a function of the number of bit errors in the soft responses. The DSR is shown for a range from 0 to 220 bit errors since beyond this range the DSR remains consistently at 0.

The resulting DSR values in Figure 8 clearly highlight the temperature dependence of the error-correction range for the considered LDPC codes. For all code configurations, higher error-correction ranges can be observed at temperatures of 10 °C and 35 °C compared to 0 °C and 50 °C. This effect can be explained by using the CRPs at $T_{ref}=20$ °C as the reference, as discussed in Section 3.1.2. Regarding bit stability, the number of unstable bits ($\mu_{count}^{u}$, $\sigma_{count}^{u}$) and their magnitudes ($\mu_{LLR}^{u}$, $\sigma_{LLR}^{u}$) for the 2-4 DAPUF design in Table 3 are similar at the considered temperatures and therefore do not directly explain these differences in DSR. Our previous reliability analysis [20] showed that differences in bit-stability characteristics can affect error-correction performance; however, their influence is comparatively limited because unstable bits are not considered bit errors in the soft responses. The corresponding bit-error parameters ($\mu_{count}^{e}$, $\sigma_{count}^{e}$) indicate a lower average number of bit errors with comparatively low LLR magnitudes ($\mu_{LLR}^{e}$, $\sigma_{LLR}^{e}$) at 10 °C and 35 °C. In particular, at 10 °C, the LLR magnitude of bit errors is generally lower than at 50 °C. At 50 °C, the standard deviation of the LLR magnitudes doubles, indicating a broader distribution and an increased likelihood of strong bit errors compared with 10 °C. Consequently, the impact of these strong bit errors becomes more pronounced as the number of introduced bit errors per measurement increases. This means that PUF reliability is high at temperatures close to $T_{ref}$, reaching a maximum at $T_{ref}$ [18]. This is also consistent with our previous comprehensive reliability analysis in [20], where the evaluated LDPC codes achieve improved PUF reliability at 10 °C and 35 °C compared to 0 °C and 50 °C. To this end, the differences between the maximum numbers of errors $N_{error}^{max}$ that can be corrected with a DSR of 1 (100%) at 10 °C and 50 °C are 25, 17, 13, 35, and 40 for the code configurations $W_{1/2}^{576}$, $W_{2/3}^{576}$, $W_{3/4}^{576}$, $W_{1/2}^{768}$, and $W_{1/2}^{864}$, respectively. Relative to the corresponding information length *K*, these differences correspond to approximately 8.68, 4.43, 3.01, 9.11, and 9.26 percentage points for the $W_{1/2}^{576}$, $W_{2/3}^{576}$, $W_{3/4}^{576}$, $W_{1/2}^{768}$, and $W_{1/2}^{864}$ codes, respectively.

As previously discussed, lower-rate ECCs typically provide higher error-correction performance, which is clearly highlighted in the resulting DSR values in Figure 8. In particular, for codes with the same information length *K*, such as $W_{2/3}^{576}$ and $W_{1/2}^{768}$ with K=384 (Figure 8, middle), as well as $W_{3/4}^{576}$ and $W_{1/2}^{864}$ with K=432 (Figure 8, bottom), higher error-correction performance can be observed for lower code rates (Table 6). As an example, at 10 °C, the $W_{1/2}^{768}$ code can correct up to $N_{error}^{max}=143$ errors with a DSR of 1, while the $W_{2/3}^{576}$ code can correct a maximum of $N_{error}^{max}=58$ errors in the soft responses. Thus, reducing the code rate from 2/3 to 1/2 results in the correction of 85 additional errors. This means that, for a PUF with a response length of K=384, responses with reliabilities of 84.90% and 62.76% can be corrected to 100% using the $W_{2/3}^{576}$ and $W_{1/2}^{768}$ codes, respectively. Such error-correction capabilities demonstrate, on the one hand, the potential to achieve highly reliable and robust PUF responses even in the presence of aging effects [27] or targeted electromagnetic fault injection attacks [50] that influence PUF behavior. On the other hand, they may pose a vulnerability since, with only approximately 62.76% of the soft-response bits being correct, the remaining incorrect soft-response bits (37.24%) could potentially be corrected by an attacker. This may indicate a security risk since a reliability of 50% corresponds to random response-generation characteristics. In this case, a successful reconstruction of the response would depend on the uniqueness characteristics of the PUF, which are investigated in the following. For the $W_{3/4}^{576}$ and $W_{1/2}^{864}$ codes, the difference at 10 °C is 129 errors. In this case, PUF responses with reliabilities of 92.13% and 62.27% can be corrected to 100% using the $W_{3/4}^{576}$ and $W_{1/2}^{864}$ codes, respectively. These significant improvements in reliability are achieved by reducing the code rate from 3/4 to 1/2. This may also indicate a potential security risk associated with the use of the lower-rate LDPC code. However, these improvements are achieved by low-rate LDPC codes, which can require high area or energy overhead depending on the code rate, number of iterations, and decoding algorithm. This overhead must be considered when implementing LDPC codes on FPGAs or ASICs with limited hardware resources [70,71,72]. For the remaining codes and temperatures, Table 6 summarizes $N_{error}^{max}$ and the corresponding minimum reliability $Q_{rel}^{pre}$ for which 100% reliability is achieved after LDPC decoding.

After evaluating the error-correction performance of different code configurations, the overall probability of an attacker successfully reconstructing the soft response, $P_{success}\left(T\right)$, can be calculated by multiplying the approximate probability of each possible $HD_{inter}$ value at temperature *T*, $P_{K}^{T}\left(HD_{inter}=i\right)$, by the corresponding DSR value at temperature *T* and summing the resulting products over all possible values of *i* from 0 to *K*, as shown in Equation (7). Consequently, the probability of successful response reconstruction by an attacker depends on two temperature-dependent characteristics: the uniqueness of the implemented PUF, represented by the $HD_{inter}$ probability distribution $P_{K}^{T}$, and the error-correction performance of the applied LDPC code for an information length of *K*, represented by the decoding success rate $DSR_{K}^{T}\left(\mu_{count}^{e}=i\right)$.

(7) $$P_{success}\left(T\right)=\sum_{i=0}^{K}P_{K}^{T}\;\left(HD_{inter}=i\right)\cdot DSR_{K}^{T}\left(\mu_{count}^{e}=i\right)$$

The resulting $P_{success}$ values for the considered LDPC codes and temperatures for the device-cloning attack, under the assumptions described in Section 3.1.3 for the simulated 2-4 DAPUF, are shown in Table 7.

The resulting $P_{success}$ values in Table 7 clearly highlight the security risks associated with low-rate LDPC codes in the considered device-cloning attack scenario. For all considered LDPC codes, the highest probability is achieved at a temperature of 10 °C, followed by 35 °C, 0 °C, and 50 °C. The greatest impact on these results arises from the high error-correction capability of the LDPC codes, as the uniqueness metric of the 2-4 DAPUF varies only slightly, by approximately 0.11 percentage points on average over the evaluated temperature range, as comprehensively investigated in [18], leading to only slight changes in the $HD_{inter}$ distribution $P_{K}^{T}$. Since the $HD_{inter}$ distribution varies only slightly across the considered temperatures, this increased error-correction capability at 10 °C explains the higher $P_{success}$ compared with 0 °C and 50 °C. For example, for $W_{1/2}^{576}$, up to 104 bit errors can be corrected with a DSR of 1 at 10 °C, compared with only 89 bit errors at 0 °C and 79 at 50 °C.

The results for the codes with a code rate of 1/2 clearly highlight significant security risks. To this end, the probability $P_{success}$ for $W_{1/2}^{576}$ at 10 °C is 62.98%, which decreases by 59.58 percentage points to 3.40% at a temperature of 50 °C. When comparing the $W_{2/3}^{576}$ and $W_{1/2}^{768}$ codes, the significant security risks associated with low-rate LDPC codes can be clearly observed. For the higher code rate $W_{2/3}^{576}$, the maximum probability of successful response reconstruction by an attacker is $2.08\cdot 10^{-8}$%, which increases to 61.82% when reducing the code rate from 2/3 to 1/2. Similar results can be observed when comparing the $W_{3/4}^{576}$ and $W_{1/2}^{864}$ codes. Here, the maximum probability increases from $4.38\cdot 10^{-21}$% to 62.87% when reducing the code rate from 3/4 to 1/2. On the other hand, using more parity information and thus a lower code rate increases the error-correction performance, as previously presented in Table 6. This clearly demonstrates a critical trade-off between PUF reliability and security. The initial investigation of information leakage from the parity information in Section 4.1 further supports the potential security risks associated with low-rate LDPC codes. As a result, under the proposed device-cloning attack scenario and the model assumptions presented in Section 3, the parity information associated with low-rate LDPC codes can facilitate response reconstruction by an attacker when the required challenge and parity information are obtained. However, this risk can be significantly reduced by using higher-rate LDPC codes. This would reduce the error-correction capability.

This investigation clearly shows that the inherent PUF reliability and uniqueness characteristics are crucial for security. This means that, for a PUF design with high and robust reliability across different conditions, higher-rate LDPC codes can be used to improve reliability while reducing the probability of a successful attack. In addition, improved uniqueness characteristics would also significantly reduce attack probabilities and should therefore be investigated. Furthermore, this investigation provides an additional criterion for ECC selection when comparing ECCs that have the same soft-information length *K* and can improve PUF reliability to 100%, alongside implementation area and energy efficiency [70,71,72].

### 4.3. Security Evaluation of the Reverse-Engineering Attack

The security evaluation of the second threat model in Figure 7 is also performed in two steps, similar to the evaluation of the first threat model. In the first step, the DSR values are evaluated for all the considered LDPC codes based on the assumptions in the second threat model. Then, the success probability, $P_{success}$, is determined.

As previously mentioned, the main difference between the two threat models is the use of responses that are not associated with a PUF implementation in the second model. Therefore, these responses provide limited or no relevant information about bit-stability and bit-error characteristics, which represent important additional information in soft responses that enables high error-correction performance, as demonstrated by the results of the first threat model. Consequently, unlike in the first attack scenario, the error-correction performance in this threat model does not depend on the ambient temperature. For the evaluation, a total of 5,000 error-free responses are generated, and a fixed number of bit errors, $\mu_{count}^{e}$ ($\sigma_{count}^{e}=0$), are introduced into each response. The remaining statistical parameters (Table 3) are therefore set to 0. The number of introduced fixed bit errors is evaluated from 0 to *K*. The resulting LP soft inputs are subsequently used by the attacker in combination with the parity information *p* to decode and thus reconstruct the secret response. Since the soft information for each bit in the soft responses is clipped to either $+L_{max}$ or $-L_{max}$, a bit error represents the strongest possible error.

**Figure 9:**
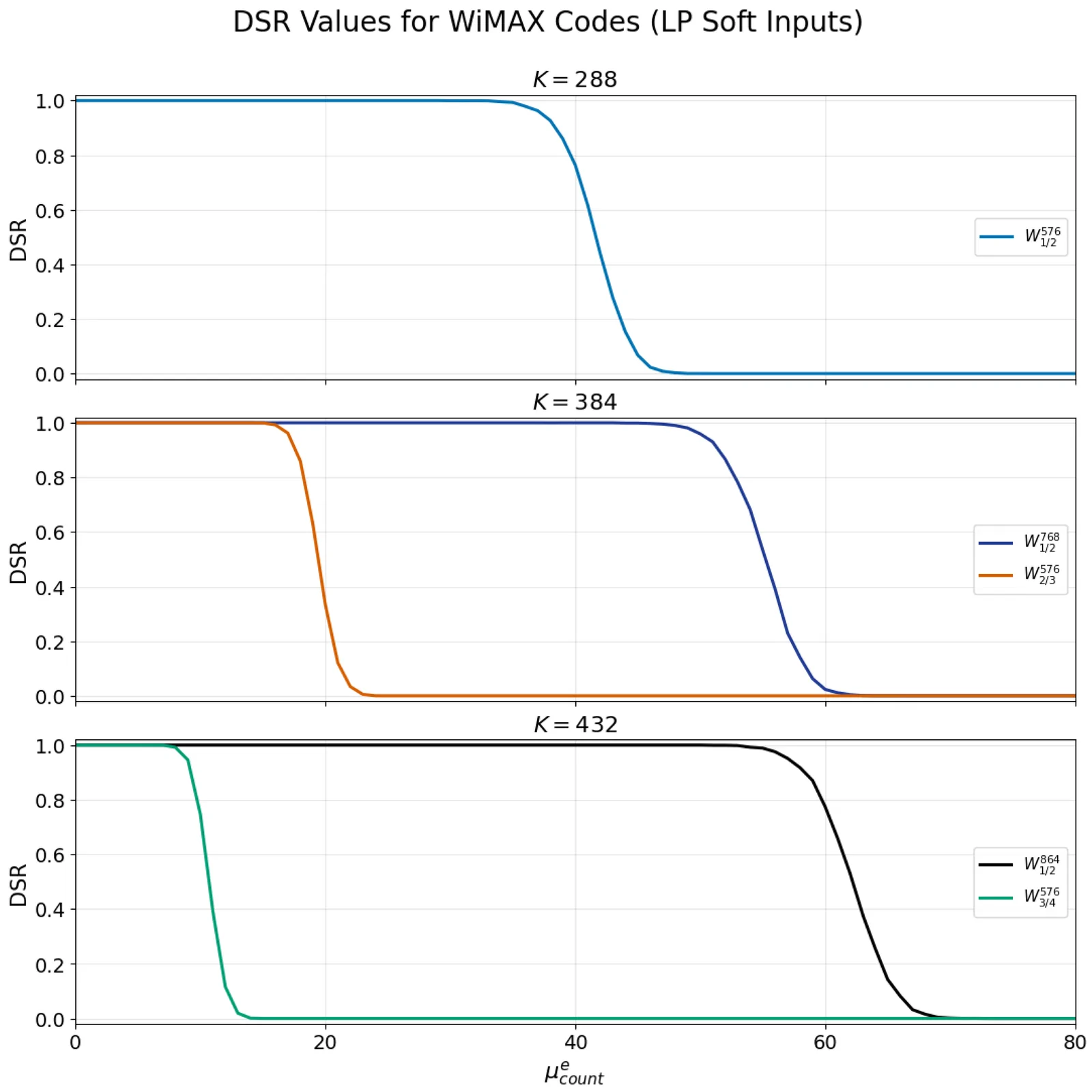
Decoding success rate (DSR) as a function of the number of bit errors $\mu_{count}^{e}$ in the LP soft responses using the considered WiMAX codes for the second threat model.

In Figure 9, the DSR evaluation for all LDPC codes with LP soft inputs as a function of the number of fixed bit errors is presented. By comparing the DSR values with those in Figure 8, the improvements in error-correction performance using soft inputs instead of LP soft inputs can be clearly observed. To this end, for the $W_{1/2}^{576}$ code, the maximum number of corrected errors for a DSR of 1 is $N_{error}^{max}=29$, while the corresponding values for soft inputs range from 79 to 104 errors over the evaluated temperature range (Table 6). In addition, for the $W_{2/3}^{576}$ code, the maximum number of corrected errors is $N_{error}^{max}=13$ (DSR=1), while for the lower code rate $W_{1/2}^{768}$, this value increases to $N_{error}^{max}=43$, leading to the correction of 25 additional errors. Similarly, compared to the soft inputs, the error-correction performance is significantly reduced for LP soft inputs. The lowest $N_{error}^{max}$ value of 6 is obtained for $W_{3/4}^{576}$, which is improved to 50 for $W_{1/2}^{864}$. The reduced error-correction capabilities can be explained by the absolute maximum LLR magnitude $L_{max}$ assigned to bit errors in the LP soft responses. Due to these high LLR magnitudes, the decoder is not able to correct a large number of errors in the LP soft responses.

Based on the model-based results of the device-cloning attack scenario, the reduced DSR values in Figure 9 initially indicate that the resulting probabilities for the reverse-engineering attack scenario can be expected to be significantly lower compared to those of the first attack scenario.

For the determination of $P(HD_{inter}=\mu_{count}^{e})$, the probability of generating a random response with an $HD_{inter}$ equal to $\mu_{count}^{e}$ compared to the secret response ($HD_{inter}=\mu_{count}^{e}=HD\left(R_{secret},R_{secret}^{\prime }\right)$) is evaluated. In the following, the probability $P_{success}$ is investigated for both cases, considering both independent and correlated bit differences (Section 3.2.2).

#### 4.3.1. Independent Bit Differences

In this case, it is assumed that each bit of the generated random response independently differs from the corresponding bit of the secret response with a probability of *q*. Here, q=0.50 represents a fully random attacker response, for which 50% of the bits differ from the secret response on average. This corresponds to the assumption that the attacker has no information about potential offsets of the victim’s PUF caused by non-symmetric structures and routing configurations [20,53]. Furthermore, it is assumed that the attacker obtains limited statistical information regarding potential offset characteristics of the PUF responses, which increases the probability that the generated response bits match the corresponding bits of the secret response. To simulate varying degrees of such information, $P(HD_{inter}=\mu_{count}^{e})$ is evaluated for q∈{0.50,0.45,0.40,0.35,0.30}. For q<0.50, the generated random response has a higher probability of matching the corresponding bits of the secret response. For the lowest investigated value, q=0.30, only 30% of the response bits differ from the secret response on average, corresponding to approximately 86, 115, and 130 bit errors for K=288, 384, and 432, respectively. The probability of generating a random response of length *K* with exactly $\mu_{count}^{e}$ bit errors can be calculated using the probability mass function of the binomial distribution, according to Equation (8).

(8) P(HDinter=μcounte)=Kμcounteqμcounte(1−q)K−μcounte

Finally, $P_{success}$ for all considered LDPC codes can be calculated by multiplying the probability $P(HD_{inter}=\mu_{count}^{e})$ by the corresponding DSR for $\mu_{count}^{e}$ bit errors and summing the resulting products over all possible values from 0 to *K*, as in the first threat model, according to Equation (9).

(9) $$P_{success}=\sum_{i=0}^{K}P_{K}\;\left(HD_{inter}=i\right)\cdot DSR_{K}\left(\mu_{count}^{e}=i\right)$$

The resulting $P_{success}$ values for the LDPC codes are shown in Table 8. Here, it can be observed that the probability of successful reconstruction from random responses (q=0.50) is effectively zero. However, if the attacker has relevant statistical information, for example, regarding potential routing-induced offsets (Figure 1, blue and red), the attacker-generated responses can contain a larger number of bits that match the corresponding bits of the secret response. For the worst-case scenario considered in this analysis, q=0.30, where 30% of the attacker-generated response bits differ from the corresponding bits of the secret response, the maximum $P_{success}$ values of $2.73\cdot 10^{-7}$%, $5.34\cdot 10^{-10}$%, and $2.95\cdot 10^{-11}$% are obtained for $W_{1/2}^{576}$, $W_{1/2}^{768}$, and $W_{1/2}^{864}$, respectively, which are still extremely low. For the remaining codes, these $P_{success}$ values are further reduced due to their lower error-correction performance.

Overall, considering the results in Section 4.1, it can be observed that the parity information of low-rate LDPC codes leaks significant information. However, the results shown in Table 8 demonstrate that, under the assumed conditions of this threat model, successful reconstruction of the secret response remains highly unlikely and therefore does not pose a significant vulnerability compared with the device-cloning attack.

#### 4.3.2. Correlated Bit Differences

In addition to considering different probabilities *q* of individual bit differences between the two responses, the influence of correlations between bit differences is investigated. The previously considered binomial distribution assumes that the bit differences between the response generated by the attacker and the secret response occur independently. However, correlations between individual bit differences may affect the resulting $HD_{inter}$ distribution and consequently the probability of successful response reconstruction. Similar to the first case for q<0.50, structural, routing, or asymmetric routing characteristics of the PUF could provide additional statistical information to the attacker. In this case, their potential influence is investigated by considering correlations between individual bit differences. To investigate this effect, $P_{success}$ is evaluated as a function of the correlation coefficient ρ. To isolate the influence of the correlation, *q* is kept constant at 0.50, while ρ∈{0,0.001,0.005,0.01} is considered. These ρ values are used as sensitivity parameters and do not represent experimentally measured correlation coefficients of the implemented 2-4 DAPUF. Here, ρ=0 represents statistically independent bit differences, corresponding to the previously considered binomial distribution. In contrast, ρ=0.01 represents a weak positive pairwise correlation, meaning that the occurrence of a bit difference slightly increases the probability that additional bit differences occur at other bit positions between the same pair of responses.

For the evaluated ρ values with 0<ρ<1, the $HD_{inter}$ distribution can be modeled using a beta-binomial distribution, according to Equation (10), which extends the previously considered binomial distribution by allowing positive correlations between the individual bit differences.

(10) PKρ(HDinter=i)=KiB(i+α,K−i+β)B(α,β)α=q1ρ−1,β=(1−q)1ρ−1

Similar to the previous analysis, $P_{success}$ is calculated by combining the resulting $HD_{inter}$ distribution $P_{K}^{\rho }\left(HD_{inter}=i\right)$ with the corresponding DSR values according to Equation (9). Here, it should be noted that for ρ=0, Equation (8) is used instead, since otherwise a division by zero would occur.

The resulting $P_{success}$ values for the investigated correlation coefficients ρ and the considered WiMAX codes are shown in Table 9.

The probability values in Table 9 show that $P_{success}$ increases with increasing ρ for all investigated WiMAX codes. This can be explained by the increasing variance of the $HD_{inter}$ distribution for positively correlated bit differences. As a result, compared to the previous case, lower and higher $HD_{inter}$ values occur with increased probability. In particular, responses with sufficiently low $HD_{inter}$ values can therefore be corrected by the LDPC codes, resulting in higher $P_{success}$ values. For ρ=0.01, the highest $P_{success}$ values of $8.43\cdot 10^{-10}\%$, $8.29\cdot 10^{-11}\%$, and $3.86\cdot 10^{-11}\%$ are obtained for the LDPC codes with a code rate of 1/2. The remaining codes show $P_{success}$ values of $2.73\cdot 10^{-22}\%$ and $2.37\cdot 10^{-29}\%$ for the $W_{2/3}^{576}$ and $W_{3/4}^{576}$ codes, respectively. Similar to the first case for q<0.50, additional information regarding the PUF structure, routing, or asymmetric routing behavior could enable the attacker to generate responses with these characteristics. However, under the considered correlation model and investigated ρ values, the reverse-engineering attack does not pose a significant vulnerability.

## 5. Conclusions and Outlook

This paper quantitatively investigated potential security risks associated with low-rate WiMAX LDPC codes in PUF-based applications using a simulated 2-4 DAPUF. To this end, a device-cloning attack and a reverse-engineering attack, representing typical attack vectors on PUF-integrated devices, were evaluated, and the probability of successful reconstruction of the secret response was determined for both threat models. Under the model assumptions, the results demonstrate that the combination of the considered PUF model with low-rate LDPC codes can significantly improve reliability; however, this combination can introduce a trade-off between PUF reliability and security.

The model-based quantitative results for the 2-4 DAPUF and the considered WiMAX LDPC codes highlight the importance of ECC selection under the considered threat models and model assumptions. Under these conditions, a significant security risk was observed for the device-cloning attack. Regarding the information leakage from the parity information, the results of the rank-based analysis indicate that the considered low-rate LDPC codes can reveal crucial information about the encoded response. Since this information is security-relevant in the proposed system model, the results clearly show that the parity information should be protected accordingly. However, it was shown that this potential security risk may be significantly reduced by using higher-rate LDPC codes, as highlighted by the number of response candidates with the same parity information, which significantly increases from 16 to $2^{192}$ for $W_{1/2}^{768}$ and $W_{2/3}^{576}$, respectively. In addition, an increase from 256 to $2^{289}$ response candidates was determined for $W_{1/2}^{864}$ and $W_{3/4}^{576}$, respectively. For the device-cloning attack scenario and the evaluation of the DSR values for soft inputs, it was evident that superior error-correction performance was achieved for lower code rates compared to higher code rates. However, regarding the probabilities of successful response reconstruction, several significant aspects could be highlighted. Due to the temperature dependence of PUF behavior, the $P_{success}$ values significantly varied from 3.40% to a maximum of 62.98%, from 1.52% to 61.82%, and from 1.11% to 62.87% for the $W_{1/2}^{576}$, $W_{1/2}^{768}$, and $W_{1/2}^{864}$ codes, respectively. This clearly demonstrates that developing PUFs that are robust against environmental conditions and integrating measures to monitor such variations are essential. These probability values additionally indicate significant security risks associated with the considered low-rate WiMAX LDPC codes under the device-cloning attack threat model. However, the results showed that these probabilities could be significantly reduced by using higher code rates. For the reverse-engineering attack, on the other hand, under the considered assumptions and for the investigated sensitivity parameters, the resulting attack success probabilities remain extremely low. This indicates the importance of PUFs because, as in this attack scenario, the PUF as well as its behavior was unavailable to the attacker, resulting in extremely low probabilities.

Overall, for the evaluated 2-4 DAPUF model and the considered WiMAX LDPC codes, the quantitative results indicate that the inherent PUF metrics of reliability and uniqueness are important factors affecting the resulting attack success probabilities under the considered threat models and model assumptions. Accordingly, high-rate codes using soft inputs can demonstrate good error-correction capabilities, but these are suitable for PUFs with high inherent reliability. Regarding uniqueness, a PUF with ideal uniqueness characteristics (50%) would also significantly reduce the investigated probabilities, as these codes are not able to reconstruct a previously generated response from random responses. In addition, the importance of further security measures, such as bitstream encryption, is also highlighted. Such measures may reduce the risk of cloning the hardware configuration on a second device.

However, this work also has several limitations. First, the quantitative security analysis is limited to the investigated PUF model, the considered WiMAX code configurations, the two threat models, and the underlying model assumptions. Second, the presented device-cloning and reverse-engineering attack scenarios have not yet been experimentally validated using a complete hardware system, such as an FPGA- or ASIC-based implementation. Third, the reliability and security analyses were performed under temperature variations. Considering additional operating conditions, such as varying supply voltage, aging effects, or electromagnetic interference, would further strengthen the evaluation.

Our next research step will focus on the hardware implementation and evaluation of a PUF on an ASIC to assess various influencing factors, optimization techniques, and security aspects. In addition, it is planned to develop a PUF-based application as a real-world use case. In this context, we plan to define guidelines for the practical deployment of PUFs in security applications, which may further support and motivate their integration into devices as lightweight security primitives. Furthermore, the planned security analysis will be extended to additional ECC mechanisms and code configurations. This will allow us to investigate how different error-correction characteristics affect the security of PUF-based applications. Based on these investigations, suitable ECC configurations can be identified depending on the PUF performance and the requirements of the corresponding application.

## Figures and Tables

**Table 1:** Overview of PUF reliability analyses under variations in supply voltage (V) and ambient temperature (T) reported in related work.

Work	PUF Design	Operating Condition	$Q_{\mathrm{rel}}^{\min}$	$Q_{\mathrm{rel}}^{\max}$	$Q_{\mathrm{rel}}$
[45]	FF-APUF	V	87.50%	98%	93.90%
	APUF	V	94%	100%	97.03%
[46]	DL-PUF	-	-	-	99.62%
[47]	TERO-PUF	T+V	85%	-	97.40%
[48]	Anderson PUF	V	68.29%	98.51%	85.41%
[49]	PDL-APUF	-	-	-	95.93%
[32]	2-2 DAPUF	T	93.30%	96.63%	95.16%
	2-2 XOR-DAPUF	T	87.60%	93.52%	90.91%
[18]	2-4 DAPUF	T	96.73%	97.88%	97.25%
	2-4 XOR-DAPUF	T	-	-	95.85%

**Table 2:** Overview of the codeword, information, and parity lengths *N*, *K*, and *P*, respectively, for the WiMAX codes considered in this security analysis, with code rates of 1/2, 2/3, and 3/4, adapted from [57].

Code	Rate	*N*	*K*	*P*
$W_{1/2}^{576}$	1/2	576	288	288
$W_{2/3}^{576}$	2/3	576	384	192
$W_{3/4}^{576}$	3/4	576	432	144
$W_{1/2}^{768}$	1/2	768	384	384
$W_{1/2}^{864}$	1/2	864	432	432

**Table 3:** Evaluated statistical parameters of the 2-4 DAPUF design [20].

Temp. $T_{x}$ ($T_{\mathrm{ref}}=$ 20 °C)	Unstable Bit		Bit Error	
	Count $\mu_{\mathrm{count}}^{u}\left(\sigma_{\mathrm{count}}^{u}\right)$	Magnitude $\mu_{\mathrm{LLR}}^{u}\left(\sigma_{\mathrm{LLR}}^{u}\right)$	Count $\mu_{\mathrm{count}}^{e}\left(\sigma_{\mathrm{count}}^{e}\right)$	Magnitude $\mu_{\mathrm{LLR}}^{e}\left(\sigma_{\mathrm{LLR}}^{e}\right)$
0 °C	3.527 (1.767)	0.075 (2.034)	1.613 (0.863)	−0.282 (2.072)
10 °C	3.747 (1.842)	0.054 (2.025)	1.452 (0.718)	−0.17 (1.326)
35 °C	3.841 (1.869)	0.031 (2.028)	1.52 (0.781)	−0.027 (1.549)
50 °C	3.674 (1.816)	0.025 (2.035)	1.817 (1.037)	0.034 (2.675)

**Table 4:** Overview of the statistical models and probability distributions used in the security analysis.

Quantity	Description	Statistical Approach
U,E	Number of unstable bits and bit errors in the simulated soft responses	Gaussian distributions with empirically obtained temperature-dependent count parameters
LLR values	Magnitudes of unstable bits and bit errors in the simulated soft responses	Gaussian distributions with empirically obtained temperature-dependent LLR parameters
$P_{K}^{T}\left(HD_{\mathrm{inter}}\right)$	Inter-device HD distribution in the device-cloning attack	Empirical discrete probability distribution extrapolated to *K* by convolution
$P_{K}\left(HD_{\mathrm{inter}}=i\right)$	HD distribution between the attacker-generated response and the secret response in the reverse-engineering attack	Binomial distribution assuming statistically independent bit differences with the same probability *q* at each bit position, where *q* is used as a sensitivity parameter
$P_{K}^{\rho }\left(HD_{\mathrm{inter}}=i\right)$	HD distribution between the attacker-generated response and the secret response with positively correlated bit differences in the reverse-engineering attack	Beta-binomial distribution with q=0.50 and positive pairwise correlation ρ, where ρ is used as a sensitivity parameter

**Table 5:** Results of the rank-based information leakage analysis of the parity information for the considered WiMAX codes.

Code	Rate	*N*	*K*	*P*	Rank(A)	*d*	$N_{\mathrm{responses}}$
$W_{1/2}^{576}$	1/2	576	288	288	283	5	32
$W_{2/3}^{576}$	2/3	576	384	192	192	192	$2^{192}$
$W_{3/4}^{576}$	3/4	576	432	144	143	289	$2^{289}$
$W_{1/2}^{768}$	1/2	768	384	384	380	4	16
$W_{1/2}^{864}$	1/2	864	432	432	424	8	256

**Table 6:** Overview of the maximum number of bit errors ($N_{error}^{max}$) that can be corrected with a DSR of 1 at temperature *T* and the corresponding reliability for the WiMAX codes with code rates of 1/2, 2/3, and 3/4.

Code	Temp. *T*	$N_{\mathrm{error}}^{\max}$	$Q_{\mathrm{rel}}^{\mathrm{pre}}$
$W_{1/2}^{576}$ (K=288)	0 °C	89	69.10%
	10 °C	104	63.89%
	35 °C	98	65.97%
	50 °C	79	72.57%
$W_{2/3}^{576}$ (K=384)	0 °C	47	87.76%
	10 °C	58	84.90%
	35 °C	55	85.68%
	50 °C	41	89.32%
$W_{3/4}^{576}$ (K=432)	0 °C	24	94.44%
	10 °C	34	92.13%
	35 °C	32	92.59%
	50 °C	21	95.14%
$W_{1/2}^{768}$ (K=384)	0 °C	121	68.49%
	10 °C	143	62.76%
	35 °C	134	65.10%
	50 °C	108	71.88%
$W_{1/2}^{864}$ (K=432)	0 °C	137	68.29%
	10 °C	163	62.27%
	35 °C	154	64.35%
	50 °C	123	71.53%

**Table 7:** Resulting $P_{success}$ values for the investigated WiMAX code configurations of the first threat model.

Code	Temp. *T*	$P_{\mathrm{success}}$
$W_{1/2}^{576}$ (K=288)	0 °C	16.31%
	10 °C	62.98%
	35 °C	46.32%
	50 °C	3.40%
$W_{2/3}^{576}$ (K=384)	0 °C	1.15 $\cdot \;10^{-11}$%
	10 °C	2.08 $\cdot \;10^{-8}$%
	35 °C	2.01 $\cdot \;10^{-9}$%
	50 °C	5.57 $\cdot \;10^{-14}$%
$W_{3/4}^{576}$ (K=432)	0 °C	7.20 $\cdot \;10^{-25}$%
	10 °C	4.38 $\cdot \;10^{-21}$%
	35 °C	1.33 $\cdot \;10^{-21}$%
	50 °C	2.55 $\cdot \;10^{-27}$%
$W_{1/2}^{768}$ (K=384)	0 °C	11.42%
	10 °C	61.82%
	35 °C	42.82%
	50 °C	1.52%
$W_{1/2}^{864}$ (K=432)	0 °C	10.19%
	10 °C	62.87%
	35 °C	42.70%
	50 °C	1.11%

**Table 8:** Resulting $P_{success}$ values for the investigated WiMAX code configurations as a function of *q* for the second threat model.

Code	$P_{\mathrm{success}}$/%				
	q=0.50	q=0.45	q=0.40	q=0.35	q=0.30
$W_{1/2}^{576}$ (K=288)	3.39 $\cdot \;10^{-32}$	1.84 $\cdot \;10^{-24}$	8.77 $\cdot \;10^{-18}$	4.51 $\cdot \;10^{-12}$	2.73 $\cdot \;10^{-7}$
$W_{2/3}^{576}$ (K=384)	1.53 $\cdot \;10^{-79}$	1.21 $\cdot \;10^{-65}$	3.74 $\cdot \;10^{-53}$	6.78 $\cdot \;10^{-42}$	9.51 $\cdot \;10^{-32}$
$W_{3/4}^{576}$ (K=432)	1.30 $\cdot \;10^{-105}$	6.63 $\cdot \;10^{-89}$	9.02 $\cdot \;10^{-74}$	5.50 $\cdot \;10^{-60}$	2.20 $\cdot \;10^{-47}$
$W_{1/2}^{768}$ (K=384)	2.77 $\cdot \;10^{-43}$	6.15 $\cdot \;10^{-33}$	5.14 $\cdot \;10^{-24}$	2.18 $\cdot \;10^{-16}$	5.34 $\cdot \;10^{-10}$
$W_{1/2}^{864}$ (K=432)	8.89 $\cdot \;10^{-49}$	4.36 $\cdot \;10^{-37}$	5.07 $\cdot \;10^{-27}$	1.96 $\cdot \;10^{-18}$	2.95 $\cdot \;10^{-11}$

**Table 9:** Resulting $P_{success}$ values for the investigated WiMAX code configurations and correlation coefficients ρ with q=0.50 for the second threat model.

Code	$P_{\mathrm{success}}$/%			
	ρ=0	ρ=0.001	ρ=0.005	ρ=0.01
$W_{1/2}^{576}$ (K=288)	3.39 $\cdot \;10^{-32}$	5.57 $\cdot \;10^{-26}$	5.84 $\cdot \;10^{-15}$	8.43 $\cdot \;10^{-10}$
$W_{2/3}^{576}$ (K=384)	1.53 $\cdot \;10^{-79}$	1.99 $\cdot \;10^{-61}$	5.82 $\cdot \;10^{-34}$	2.73 $\cdot \;10^{-22}$
$W_{3/4}^{576}$ (K=432)	1.30 $\cdot \;10^{-105}$	2.07 $\cdot \;10^{-80}$	2.63 $\cdot \;10^{-44}$	2.37 $\cdot \;10^{-29}$
$W_{1/2}^{768}$ (K=384)	2.77 $\cdot \;10^{-43}$	7.01 $\cdot \;10^{-33}$	3.27 $\cdot \;10^{-17}$	8.29 $\cdot \;10^{-11}$
$W_{1/2}^{864}$ (K=432)	8.89 $\cdot \;10^{-49}$	6.16 $\cdot \;10^{-36}$	4.84 $\cdot \;10^{-18}$	3.86 $\cdot \;10^{-11}$

## Data Availability

The datasets presented in this article are not readily available because the data are part of an ongoing study. Requests to access the datasets should be directed to the corresponding author.
